# Hydroxyl-Functionalized Covalent Organic Frameworks as High-Performance Supercapacitors

**DOI:** 10.3390/polym14163428

**Published:** 2022-08-22

**Authors:** Tzu-Ling Yang, Jhu-You Chen, Shiao-Wei Kuo, Chen-Tsyr Lo, Ahmed F. M. El-Mahdy

**Affiliations:** 1 Department of Materials and Optoelectronic Science, National Sun Yat-Sen University, Kaohsiung 80424, Taiwan; 2 Department of Material Science and Engineering, National Taiwan University of Science and Technology, Taipei 10617, Taiwan; 3 Chemistry Department, Faculty of Science, Assiut University, Assiut 71516, Egypt

**Keywords:** covalent organic frameworks, dihydroxynaphthalene, redox-active, Schiff-base, supercapacitors

## Abstract

Covalent organic frameworks (COFs) have attracted significant interest because of their heteroatom-containing architectures, high porous networks, large surface areas, and capacity to include redox-active units, which can provide good electrochemical efficiency in energy applications. In this research, we synthesized two novel hydroxy-functionalized COFs—TAPT-2,3-NA(OH)_2,_ TAPT-2,6-NA(OH)_2_ COFs—through Schiff-base [3 + 2] polycondensations of 1,3,5-tris-(4-aminophenyl)triazine (TAPT-3NH_2_) with 2,3-dihydroxynaphthalene-1,4-dicarbaldehyde (2,3-NADC) and 2,6-dihydroxynaphthalene-1,5-dicarbaldehyde (2,6-NADC), respectively. The resultant hydroxy-functionalized COFs featured high BET-specific surface areas up to 1089 m^2^ g^–1^, excellent crystallinity, and superior thermal stability up to 60.44% char yield. When used as supercapacitor electrodes, the hydroxy-functionalized COFs exhibited electrochemical redox activity due to the presence of redox-active 2,3-dihydroxynaphthalene and 2,6-dihydroxynaphthalene in their COF skeletons. The hydroxy-functionalized COFs showed specific capacitance of 271 F g^−^^1^ at a current density of 0.5 A g^−^^1^ with excellent stability after 2000 cycles of 86.5% capacitance retention. Well-known pore features and high surface areas of such COFs, together with their superior supercapacitor performance, make them suitable electrode materials for use in practical applications.

## 1. Introduction

Since the 20th century, the massive usage of fossil fuels including coal, gas, and mineral oil has been essential to economic and industrial advancement [1,2]. Renewable fuel technologies are increasingly frequently integrated with energy storage technologies to make better use of the converted energy, which can lessen the negative effects of poisonous air, global warming, and unsuitable ecosystems [3,4,5]. Energy storage forms for harvesting energy include mechanical, electrochemical, thermal, electrical, and hydrogen-based storage [6]. Among them, electrochemical supercapacitors (Scs) have received a lot of interest because they have a higher energy density, a longer life cycle, quicker storing capabilities, and a faster charge/discharge rate than ordinary dielectric capacitors [7,8,9]. A variety of electrochemical SC technologies for energy storage purposes have been created, including lithium ion batteries, sodium ion batteries, and magnesium ion batteries [10,11,12,13]. There are two types of methods for storing energy in supercapacitors: non-faradaic procedures, in which electrostatic ionic charges gather at the interface between electrolyte and electrode; and faradaic processes, which occur at the solid material’s surface via the irreversible redox reaction. Consequentially, the electrode materials must exhibit good thermal stability, accurate pore size distributions, and steady electrochemical activity [14,15,16]. In the strategy to boost their efficiency in SCs, a variety of innovative porous carbon-based materials, such as activated carbon, nano- and meso-porous carbon, graphene aerogels, and porous graphene, have been performed [17,18,19,20,21]. In addition, doping such carbon materials with nitrogen can increase the effectiveness of supercapacitors due to the pseudo-capacitance effect that nitrogen atoms provide [22,23]. Because they possess amino moieties in the framework skeletons, specific crystalline systems, tuneable porosities, and a wide availability to various types of heteroatom doping, several porous polymer materials, such as conjugated polymers, covalent organic frameworks (COFs), covalent triazine frameworks (CTFs), and hyper crosslinked polymers have been regarded as perfect materials when used as electrodes in supercapacitors [24,25,26,27]. However, because of their weak inherent conductivity, the capacitance of such polymers remains very low. As a result, novel crystalline porous polymers with redox active moiety and high intrinsic conductivity are desperately needed.

COFs are crystalline organic polymers with organized pores and periodic skeletons. COFs are comprised of light elements and are synthesized through a reversible condensation reaction that results in stable structures due to dynamic covalent chemistry [28,29,30,31,32]. Moreover, the most appealing feature of COFs is that their chemical structures could be precisely adjusted by altering their building linkers through an organic synthesis, to meet the needs of the intended application [33,34]. COFs have already found widespread applications in catalysis, optoelectronics, gas storage, separation, medication delivery, environmental cleanup, and energy storage [35,36,37,38,39]. In terms of electrochemistry, COFs have specific electrochemical characteristics over non-crystalline polymers due to their controlled pore diameters and the ability to include redox-active moieties into their frameworks by altering their building blocks [40]. In addition, the known pore properties and hierarchical topologies of COFs, together with their superior supercapacitor performance, make them appropriate electrode materials for real-world use [40]. According to recent research, the supercapacitor efficiency of COFs could be increased in two ways: (i) by inserting a redox moiety in β-ketoenamine-linked COFs [41,42,43] and (ii) by inserting metallic ions in polyimine-based COFs [44]. In the first strategy, the inclusion of a redox-active moiety—for example, 2,5-diaminopyridine, carbazole, triphenylamine, triphenylpyridine or 2,6-diaminoanthraquinone moiety—into the hexagonal backbone of the COF has indeed improved supercapacitor efficiency [41,42,43]. Although these reported examples all featured good specific capacitance, the number of redox-active moieties incorporated into COF frameworks for usage as novel electrode materials has remained restricted to such units. In the second strategy, polyimine-based COFs containing metal ions (Co^II^, Ni^II^, Fe^III^) in their cavities were pyrolyzed (900 °C, 4 h) into N-doped porous graphenes with good specific capacitances in KOH as the electrolyte, possibly making them usable as electrodes for supercapacitors [44]. Although the specific capacitances of these pyrolyzed COFs were greater than those of redox-active COFs, their production required difficult circumstances, and the resultant COFs lost their crystallinity after being completely transformed into graphene. Therefore, the development of new, highly electrochemically efficient COFs with redox-active moieties is still of interest.

Taking into account all of this above knowledge, we present in this work the utilization of the redox-active and hydroxyl-functionalized monomers—2,3-dihydroxynaphthalene-1,4-dicarbaldehyde (2,3-NADC) and 2,6-dihydroxynaphthalene-1,5-dicarbaldehyde (2,6-NADC)—to synthesize two hydroxy-functionalized COFs, namely TAPT-2,3-NA(OH)_2,_ TAPT-2,6-NA(OH)_2_ COFs, through the [3 + 2] polycondensations with1,3,5-tris-(4-aminophenyl)triazine (TAPT-3NH_2_). After that, the resultant hydroxyl-functionalized COFs were evaluated as potential supercapacitor electrodes, and their capacitive properties, such as cyclic voltammetry, galvanostatic charge/discharge, and cyclic durability, were investigated.

## 2. Materials and Methods

### 2.1. Materials

2,3-Dihydroxynaphthalene, 2,6-dihydroxynaphthalene, 4-bromoaniline, potassium carbonate, and formamidine were received from Acros. Acetic anhydride, HCl, and triflic acid were ordered from Alfa Aesar. Dioxane, chloroform, *n*-butanol and *o*-dichlorobenzene, acetic acid, DMF, THF, and acetone were obtained from J. T. Baker (Phillipsburg, NJ, USA). Raw chemical materials and solvents were purchased from commercial resources and then used as they were.

### 2.2. Synthesis of Dihydroxynaphthalene Dicarbaldehyde (NADC)

Formamidine acetate (14.98 mmol) and dioxane (30 mL) were added into a 100-mL two-neck round-bottom flask and heated under reflux. Acetic anhydride (3 mL) was added when the target temperature reached 95 °C and then stirred for 30 min. Dihydroxynaphthalene (1.87 mmol) was added once all formamidine acetate was dissolved and was kept for two days. After cooling for minutes, dioxane was evaporated at 50 °C, was added H_2_O (45 mL), and heated at 65 °C for 2 h. Then, we added HCl (1 M, 40 mL) and kept heating at 65 °C for 18 h. The solid was filtered and washed several times with hexane. Dihydroxynaphthalene dicarbaldehydes (2,3-NADC and 2,6-NADC) were obtained after purifying by using column chromatography (Schemes S2 and S3).

### 2.3. Synthesis of Hydroxyl-Functionalized Covalent Organic Frameworks

Three freeze/pump/thaw cycles were used to degas a solution of TAPT-3NH_2_ (0.196 mmol) (Scheme S1) and 2,3-NADC or 2,6-NADC (0.296 mmol) in *n*-butanol (3.5 mL) and *o*-dichlorobenzene (3.5 mL) with acetic acid (6 M, 0.7 mL) in a 25-mL Schlenk storage tube. The tube was then flame-sealed and heated at 120 °C for three days. After the tube had cooled to room temperature, the precipitate was filtered and washed (once with DMF, three times each with THF and acetone). The solid was vacuum-dried overnight at 120 °C to provide the TAPT-2,3-NA(OH)_2_ and TAPT-2,6-NA(OH)_2_ COFs (Schemes S4 and S5).

## 3. Results

### 3.1. Design, Synthesis, and Crystallinity of Hydroxyl-Functionalized COFs

**Figure 1:**
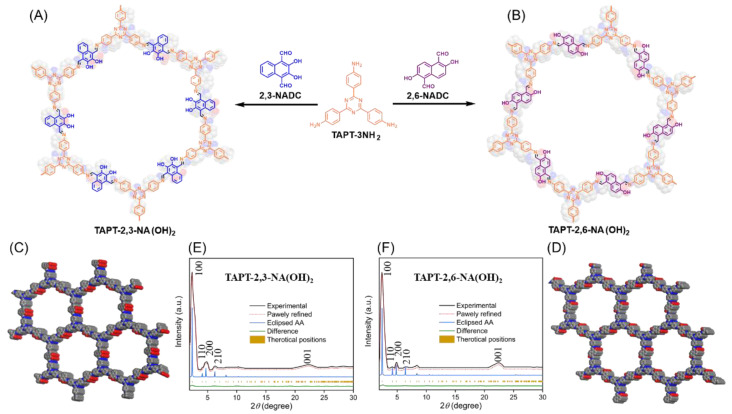
(**A**,**B**) Synthesis of (**A**) TAPT-2,3-NA(OH)_2_ and (**B**) TAPT-2,6-NA(OH)_2_ COFs. (**C**,**D**) Top-view crystal structures of (**C**) TAPT-2,3-NA(OH)_2_ and (**D**) TAPT-2,6-NA(OH)_2_ COFs. (**C**,**D**) Experimental and simulated PXRD patterns of (**E**) TAPT-2,3-NA(OH)_2_ and (**F**) TAPT-2,6-NA(OH)_2_ COFs.

To design novel hydroxyl-functionalized COFs with efficient supercapacitor performances, as well as to investigate the impact of hydroxyl group position on their characteristics and applications, we synthesized two TAPT-2,3-NA(OH)_2_ and TAPT-2,6-NA(OH)_2_ COFs–through solvothermal [3 + 2] polycondensations of TAPT-3NH_2_ (Figures S1–S3) with 2,3-NADC (Figures S4–S6) and 2,6-NADC (Figures S7–S9) as new redox-active and hydroxyl-functionalized monomers (Figure 1A,B). Their polycondensation synthesis was carried out using *n*-butanol/*o*-dichlorobenzene (1:1) as a co-solvent over 72 h at 115 °C in Schlenk tubes, in the presence of acetic acid (6 M, 10 vol%) as an acidic catalyst. In the widely-used organic solvents such as dioxane, methanol, tetrahydrofuran, DMF, acetone, or DMSO, the produced COF powders were insoluble, even at high temperatures.

We recorded powder X-ray diffraction (PXRD) patterns of the TAPT-2,3-NA(OH)_2_ and TAPT-2,6-NA(OH)_2_ COFs to gain knowledge into their crystallinities (Figure 1C–F, and Figures S10 and S11), which demonstrated that the TAPT-2,3-NA(OH)_2_ and TAPT-2,6-NA(OH)_2_ COFs both featured triclinic networks with long-ordered architectures. In Figure 1E, the experimental PXRD (black curve) of the TAPT-2,3-NA(OH)_2_ COF displayed a strong signal at 2*θ* = 2.34°, which we attribute to the 100 facet, as well as three signals at 2*θ* = 4.11°, 4.79°, and 6.24°, which we attribute to the 110, 200, and 210 facets, respectively. We ascribe the wide diffraction signal at 22.38° to the 001 facet, which emerged due to significant π-stacking between the triclinic interlayers of the TAPT-2,3-NA(OH)_2_ COF (Figure 1E and Figure S10). Similarly, TAPT-2,6-NA(OH)_2_ COF exhibited a significant signal at 2*θ* = 2.38°, which we ascribe to the 100 facet, as well as three further peaks at 2*θ* = 4.15°, 4.93°, 6.42°, and 22.45° which we assign to the 110, 200, 210, and 001 reflections, respectively (Figure 1F and Figure S11). The π-interlayer lengths between the 2D layers of the COF planes and the average *d*-spacings between the 100 planes of the hydroxyl-functionalized COFs (*d*_100_) can be calculated using the Bragg equation; for the TAPT-2,3-NA(OH)_2_ and TAPT-2,6-NA(OH)_2_ COFs, the π-interlayer lengths were 3.96 and 3.94 Å, respectively, and the values of *d*_100_ were 3.76 and 3.78 nm, respectively (Table S1). The theoretical PXRD patterns of the TAPT-2,3-NA(OH)_2_ and TAPT-2,6-NA(OH)_2_ COFs (Figure 1E,F, red-dot curves) derived from the Pawley refinements were congruent with the experimental patterns (Figure 1E,F, black curves), with only very tiny changes (Figure 1E,F, olive curves). To extensively investigate the unit cell characteristics and layer conformations of the hydroxyl-functionalized COFs, we utilized the Material Studio software to build eclipsed A-A stacking models for the TAPT-2,3-NA(OH)_2_ and TAPT-2,6-NA(OH)_2_ COFs (Figure 1E,F, blue curves, and Figures S10 and S11). The results revealed that the experimental PXRD patterns of TAPT-2,3-NA(OH)_2_ and TAPT-2,6-NA(OH)_2_ COFs both evenly matched those of the relevant AA stacking models. Importantly, the unit cells of the eclipsed AA-stacking simulations revealed the following parameters: for TAPT-2,3-NA(OH)_2_ COF, *a* = 43.4 Å, *b* = 43.2 Å, *c* = 3.649 Å, α = β = 90°, and γ = 120° (Figure S12a,b, Table S2); TAPT-2,6-NA(OH)_2_ COF, *a* = 42.3 Å, *b* = 42.1 Å, *c* = 3.5 Å, α = β = 90°, γ = 120° (Figure S12c,d, Table S3).

**Figure 2:**
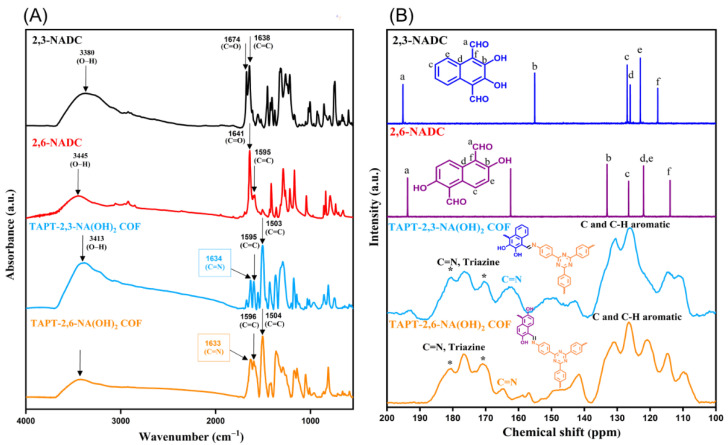
(**A**) FTIR spectra of 2,3-NADC, 2,6-NADC, TAPT-2,3-NA(OH)_2_, and TAPT-2,6-NA(OH)_2_ COFs. (**B**) ^13^C-NMR spectra of 2,3-NADC, 2,6-NADC and solid state ^13^C CP/MAS NMR spectra of TAPT-2,3-NA(OH)_2_ and TAPT-2,6-NA(OH)_2_ COFs. * Background peaks from the instrument.

The molecular structures of the hydroxyl-functionalized COFs were confirmed using FTIR and solid state ^13^C NMR spectroscopy. The FTIR spectra of TAPT-2,3-NA(OH)_2_ and TAPT-2,6-NA(OH)_2_ COFs did not show any of the distinguishable vibration signals for the amino (NH_2_) group at 13,458–3320 cm^–1^ for the TAPT-3NH_2_ monomer or the vibration signals for the aldehydic (CH=O) groups at 1674 and 1641 cm^–1^, respectively, for the 2,3-NADC and 2,6-NADC, implying complete polycondensations of TAPT-3NH_2_ with the two hydroxyl-functionalized monomers. New distinguishable vibration signals were observed at 1634 and 1633 cm^–1^ in the FTIR spectra of TAPT-2,3-NA(OH)_2_ and TAPT-2,6-NA(OH)_2_ COFs, respectively, corresponding to their imino (C=N) bonds (Figure 2A and Figures S13 and S14). The solid state ^13^C NMR spectra of the TAPT-2,3-NA(OH)_2_ and TAPT-2,6-NA(OH)_2_ COFs exhibited distinctive signals at 162.42 and 164.88 ppm for the resonances of the imino-carbon (C=N) nuclei, respectively (Figure 2B). Because of the hydrogen bond formed by the hydroxyl group and nitrogen atom of the imine group in the TAPT-2,6-NA(OH)_2_ COF, as observed in the chemical structures inside Figure 2B, the C=N nuclei of this COF are located higher than those of the TAPT-2,3-NA(OH)_2_ COF. The signals in the ranges of 138.89–107.34 and 137.75–104.89 ppm, respectively, were particularly notable for representing the aromatic-carbon (C–H and C–C) nuclei of the TAPT-2,3-NA(OH)_2_ and TAPT-2,3-NA(OH)_2_ COFs. In addition, the solid state ^13^C NMR spectra revealed signals at 176.17 and 176.65 ppm, representing the resonances of triazine units in the TAPT-2,3-NA(OH)_2_ and TAPT-2,6-NA(OH)_2_ COFs, respectively.

### 3.2. BET, XPS, TEM, FE-SEM, and TGA Analyses of Hydroxyl-Functionalized COFs

**Figure 3:**
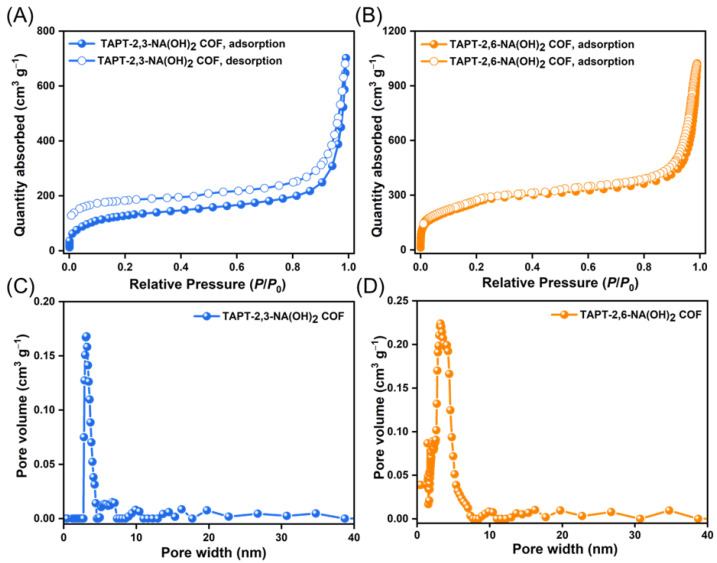
(**A**,**B**) N2 sorption isotherms recorded at 77 K of (**A**) TAPT-2,3-NA(OH)_2_ and (**B**) TAPT-2,6-NA(OH)_2_ COFs. (**C**,**D**) Pore size distributions of (**C**) TAPT-2,3-NA(OH)_2_ and (**D**) TAPT-2,6-NA(OH)_2_ COFs.

We performed an N_2_ sorption analysis at 77 K to examine the pore structures of the hydroxyl-functionalized COFs. According to the IUPAC technical report on physisorption of gases, N_2_ sorption isotherms of TAPT-2,3-NA(OH)_2_ and TAPT-2,6-NA(OH)_2_ COFs demonstrated type I isotherms, with an unremarkable N_2_ uptake in the relative pressure range (*P*/*P*_0_) less than 0.1 bar and a steep N_2_ uptake in the relative pressure range greater than 0.8 bar (Figure 3A,B). The existence of small hysteresis loops in the resulting isotherms suggested that our synthesized TAPT-2,3-NA(OH)_2_ and TAPT-2,6-NA(OH)_2_ COFs were microporous. In addition, the adsorption/desorption curves revealed that the Brunauer–Emmett–Teller (BET) surface areas of TAPT-2,3-NA(OH)_2_ and TAPT-2,6-NA(OH)_2_ COFs were 429 and 1089 m^2^ g^–1^, with pore volumes of 0.17 and 0.22 cm^3^ g^–1^, respectively (Table S1). Furthermore, we employed a non-local DFT to evaluate the pore size distribution of TAPT-2,3-NA(OH)_2_ and TAPT-2,6-NA(OH)_2_ COFs, showing pore diameters of roughly 3.04 and 3.29 nm, respectively (Figure 3C,D and Table S1).

We studied the elemental kinds and their ration in our hydroxyl-functionalized COFs using an X-ray Photoelectron Spectroscopy (XPS). As shown in Figure S15, the XPS spectra of hydroxyl-functionalized COFs revealed three distinct peaks for the carbons, nitrogens, and oxygens at 286.21, 400.61, and 533.51 eV, respectively, for the TAPT-2,3-NA(OH)_2_ COF and at 286.17, 399.61, and 533.50 eV, respectively, for theTAPT-2,6-NA(OH)_2_ COF. The lack of any additional components within XPS detections demonstrates the absence of any perceptible impurities that may have been produced during the synthesis of the hydroxyl-functionalized COFs, as illustrated in Figure S15. We fitted the XPS curves for the C1s, N1s, and O1s orbitals to better understand the kinds of N and O species found in the COFs (Figure S16). Tables S4 and S5 summarize all XPS fitting data. TAPT-2,3-NA(OH)_2_ COF has three primary kinds of C 1s species on their own surfaces: C–OH at 288.30 eV, C=C at 286.30 eV, and C=N at 284.60 eV (Figure S16a and Table S4). On the other hand, the TAPT-2,6-NA(OH)_2_ COF has also three kinds of C1s orbitals at 288.20, 286.17, and 283.45 eV, which we attributed to the C–OH, C=C, and C=N bonds, respectively (Figure S16d and Table S4). The fitting of the N1s, and O1s orbitals of both hydroxyl-functionalized COFs revealed a single kind of C1s and O1s orbitals (Figure S16b,c,e,f and Table S4) [45].

**Figure 4:**
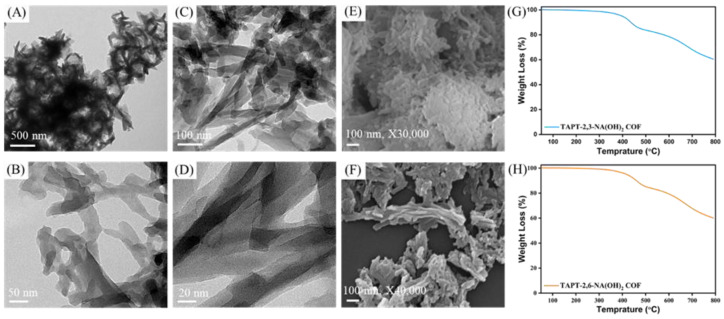
(**A**–**D**) TEM images of (**A**,**B**) TAPT-2,3-NA(OH)_2_ and (**C**,**D**) TAPT-2,6-NA(OH)_2_ COFs, measured at different magnifications. (**E**,**F**) FE-SEM images of (**E**) TAPT-2,3-NA(OH)_2_ and (**F**) TAPT-2,6-NA(OH)_2_ COFs. (**G**,**H**) TGA curves of (**G**) TAPT-2,3-NA(OH)_2_ and (**H**) TAPT-2,6-NA(OH)_2_ COFs.

We used transmission electron microscopy (TEM) and field-emission scanning electron microscopy (FE-SEM) to visualize the self-assembly morphologies of the TAPT-2,3-NA(OH)_2_ and TAPT-2,6-NA(OH)_2_ COFs (Figure 4A–F). Low-magnification TEM images of TAPT-2,3-NA(OH)_2_ and TAPT-2,6-NA(OH)_2_ COFs after solvent exfoliation in ethanol revealed that both the hydroxyl-functionalized COFs were assembled into a significant number of long nanofibers with lengths of up to several micrometers, and such nanofibers were linked by their mesoporous sidewalls (Figure 4A–D and Figures S17 and S18). The statistical analysis of the TEM images of the TAPT-2,3-NA(OH)_2_ and TAPT-2,6-NA(OH)_2_ COFs revealed average diameters for the nanofibers of 30 ± 50 and 50 ± 70 nm, respectively. As we had documented previously, the degree of planarity of the building monomers can have a significant impact on the morphology of the COFs. We also reported that the COFs constructed from planar building monomers are often built into tubes, rods, or fibers [9,46,47]. Therefore, the nanofiber morphologies of TAPT-2,3-NA(OH)_2_ and TAPT-2,6-NA(OH)_2_ COFs were apparently formed due to the high planarity of the TAPT-3NH_2_ monomer. In Figure 4E,F and Figure S19, the SEM images of the TAPT-2,3-NA(OH)_2_ and TAPT-2,6-NA(OH)_2_ COFs confirmed their nanofiber morphologies. One of the primary requirements for the possible use of porous polymers in commercial energy storage and electrochemical supercapacitor technologies is their heat stability. Both hydroxyl-functionalized COFs demonstrated exceptional thermal stability when tested using a thermogravimetric analysis (TGA) under a nitrogen environment in a temperature range from 100 to 800 °C. Figure 4G,H and Table S6 show that TAPT-2,3-NA(OH)_2_ and TAPT-2,6-NA(OH)_2_ COFs were thermally stable materials; the values of their decomposition temperatures (*T*_d10_) were 435 and 460 °C, respectively, and the char yields were 60.44 and 60.07%, respectively. The desorption of the trapped solvents could be the cause of the initial weight loss of the TAPT-2,3-NA(OH)_2_ and TAPT-2,6-NA(OH)_2_ COFs.

### 3.3. Supercapacitor Application of Hydroxyl-Functionalized COFs

**Figure 5:**
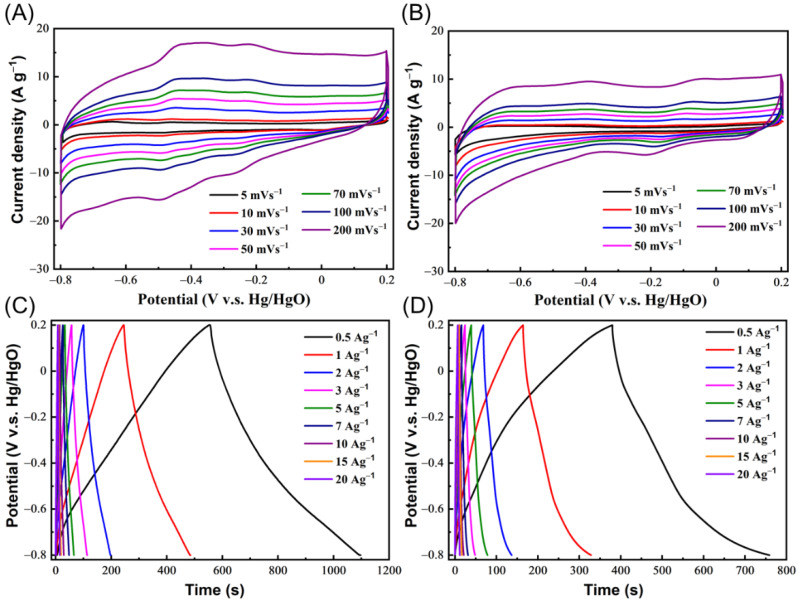
(**A**,**B**) CV curves of (**A**) TAPT-2,3-NA(OH)_2_ and (**B**) TAPT-2,6-NA(OH)_2_ COFs, measured at different scan rates (mV s^–1^). (**C**,**D**) GCD curve of (**C**) TAPT-2,3-NA(OH)_2_ and (**D**) TAPT-2,6-NA(OH)_2_ COFs, measured at different current densities (A g^−1^).

The above PXRD, BET, XPS, and TEM measurements reveal that our TAPT-2,3-NA(OH)_2_ and TAPT-2,6-NA(OH)_2_ COFs had excellent crystallinities, high surface areas, redox-active structures, and microporous frameworks, suggesting that these hydroxyl-functionalized COFs might well be employed as promising supercapacitor materials. Therefore, we evaluated the electrochemical performance of TAPT-2,3-NA(OH)_2_ and TAPT-2,6-NA(OH)_2_ COFs using cyclic voltammetry (CV) and galvanostatic charge–discharge (GCD) in a three-electrode setup with an aqueous solution of potassium hydroxide (1 M) as electrolyte. Figure 5A,B and Figure S20 reveal the CV curves of the hydroxyl-functionalized COFs in the potential window from −0.8 to +0.2 V which were recorded at various rates ranging from 5 to 200 mV s^−1^. The CV curves of TAPT-2,3-NA(OH)_2_ and TAPT-2,6-NA(OH)_2_ COFs showed quasi-rectangular shapes with small humps around −0.44 V for the TAPT-2,3-NA(OH)_2_ and around −0.39 V for the TAPT-2,6-NA(OH)_2_ COFs (Figure S21a,b), suggesting that the capacitive responses were originated mainly from the EDL capacitance and minor from pseudo-capacitance [48,49,50,51,52]. The redox reactions of 2,3-dihydroxynaphthalene or 2,6-dihydroxynaphthalene on the COF surfaces and/or the N-heteroatom functionalities of the substance can be responsible for the formation of small humps in CV curves (Figure S21c,d) [48,49,50,51,52]. The apparent peak separations between the waves of oxidation and reduction of both COFs were quite small, indicating a rapid electron transfer between the GC electrode and the dihydroxynaphthalene units in the COFs [53,54]. Additionally, the shape of the CV curves of TAPT-2,3-NA(OH)_2_ and TAPT-2,6-NA(OH)_2_ COFs were well maintained, but as the sweep rate was raised, the current density rose (Figure 5A,B), indicating a strong rate capacity and fast kinetics [55]. The GCD measurements of TAPT-2,3-NA(OH)_2_ and TAPT-2,6-NA(OH)_2_ COFs were performed in the same voltage window, but at various current densities ranging from 0.5 A g^−1^ to 20 A g^−1^. As shown in Figure 5C,D, both TAPT-2,3-NA(OH)_2_ and TAPT-2,6-NA(OH)_2_ COFs exhibited triangular-shaped GCD curves with modest bends, confirming the co-contribution of both EDLC and pseudo-capacitance, which is consistent with the CV curves. Figure 5C,D and Figure S22 show that the discharge time of the TAPT-2,3-NA(OH)_2_ COF was longer than that of the TAPT-2,6-NA(OH)_2_ COF, indicating that the capacitance of the former was greater than the latter.

**Figure 6:**
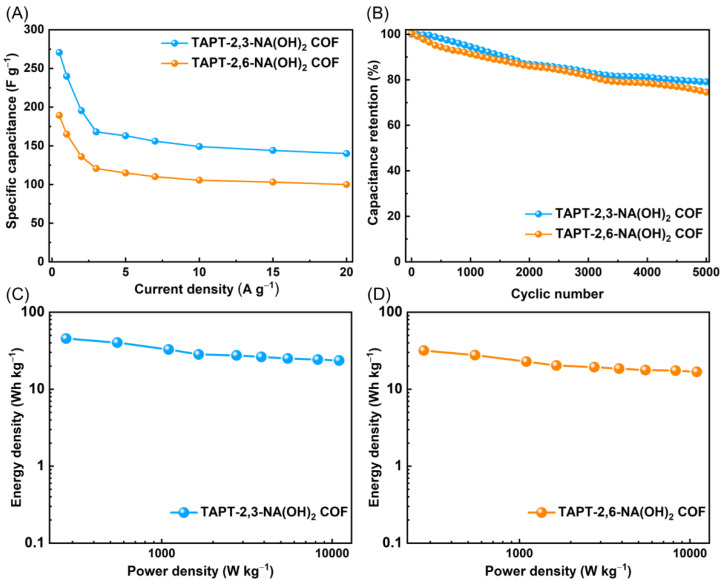
(**A**) Calculated specific capacitances of TAPT-2,3-NA(OH)_2_ and TAPT-2,6-NA(OH)_2_ COFs, measured at different current densities (A g^−1^). (**B**) Cycling performance of TAPT-2,3-NA(OH)_2_ and TAPT-2,6-NA(OH)_2_ COFs, measured at current density of 10 A g^−1^. (**C**,**D**) Ragone plot showing the energy density and power density for the (**C**) TAPT-2,3-NA(OH)_2_ and (**D**) TAPT-2,6-NA(OH)_2_ COFs electrodes.

Based on the GCD curves of our hydroxyl-functionalized COFs, we computed their specific capacitances using Equation (S1). The specific capacitance of TAPT-2,3-NA(OH)_2_ and TAPT-2,6-NA(OH)_2_ COFs can be calculated to be 271 F g^−1^ and 190 F g^−1^, respectively, at a current density of 0.5 A g^−1^ (Figure 6A). While TAPT-2,6-NA(OH)_2_ had the greatest surface area (1089 m2 g^−1^) and the largest pore volume (0.22 cm^3^ g^−1^), it had a lower specific capacitance than that of the TAPT-2,3-NA(OH)_2_. This behavior is explained by the increased redox ability of the 2,3-dihydroxynaphthalene units compared to the 2,6-dihydroxynaphthalene units. It has been reported that raising the speed of the redox reactions increased the contribution to the pseudo-capacitive behavior of the electrode and hence increased the electrochemical capacitance [55,56,57]. In addition, Table S7 reveals that the supercapacitor performances of our TAPT-2,3-NA(OH)_2_ and TAPT-2,6-NA(OH)_2_ COFs are comparable to other investigated COF materials [9,41,42,43,47,58,59]. Furthermore, we cycled our TAPT-2,3-NA(OH)_2_ and TAPT-2,6-NA(OH)_2_ COFs for up to 5000 cycles at a current density of 10 A g^−1^ to investigate their durability. As shown in Figure 6B, TAPT-2,3-NA(OH)_2_ and TAPT-2,6-NA(OH)_2_ COFs exhibited high electrochemical stabilities by maintaining 79.1 and 74.5% retentions of their initial capacitances, respectively. Furthermore, the Ragone plots in Figure 6C,D indicate that both TAPT-2,3-NA(OH)_2_ and TAPT-2,6-NA(OH)_2_ COFs had high energy and power densities; the energy density for TAPT-2,3-NA(OH)_2_ COF was found to be 45.43 Wh Kg^−1^ and for TAPT-2,6-NA(OH)_2_ COF was found to be 31.11 45.43 Wh Kg^−1^. This finding implies that these materials might be used in industrial uses.

The electrochemical impendence spectroscopy (EIS) was used to further analyze the electrochemical characteristics of our hydroxyl-functionalized COFs. Figure S23 depicted the Nyquist plots of TAPT-2,3-NA(OH)_2_ and TAPT-2,6-NA(OH)_2_ COFs with amplitude of 5 mV and in the range 0.01 Hz to 800 KHz. The semi-cycles in the high frequency domain of the Nyquist plots of TAPT-2,3-NA(OH)_2_ and TAPT-2,6-NA(OH)_2_ COFs indicated low interfacial impedance and good pore conductivity for the electrolyte ions [60]. On the other hand, our TAPT-2,3-NA(OH)_2_ and TAPT-2,6-NA(OH)_2_ COFs showed roughly vertical lines in the low frequency domain with slopes greater than 45°, indicating their strong capacitive characteristics [61]. By measuring the intersection of the Z’ axis in the high frequency domain, the intrinsic ohmic resistances (Rs), which reflected the conductivity of the hydroxyl-functionalized COFs, were calculated. The TAPT-2,3-NA(OH)_2_ COF revealed the lower value of Rs (13.4 Ω), while TAPT-2,6-NA(OH)_2_ COF showed the higher value of Rs (48.8 Ω), which consists with their specific capacitance results.

The chemical stability of TAPT-2,3-NA(OH)2 and TAPT-2,6-NA(OH)2 COFs in potassium hydroxide (1 M) was examined by soaking 30 mg of the each COF in the alkaline solution for two days, isolating it using vacuum filtration, washing it with water, and drying it at 120 °C overnight. As illustrated in Figure S24, the remarkable chemical stability of the TAPT-2,3-NA(OH)_2_ and TAPT-2,6-NA(OH)_2_ COFs in such an alkaline solution was indicated by the maintenance of FTIR signals with non-significant change after soaking in a potassium hydroxide (1 M) solution.

## 4. Conclusions

In summary, two hydroxy-functionalized COFs, namely, TAPT-2,3-NA(OH)_2,_ TAPT-2,6-NA(OH)_2_ COFs, were synthesized through the Schiff-base [3 + 2] polycondensations of TAPT-3NH_2_ with 2,3-NADC and 2,6-NADC, respectively, using *n*-butanol/*o*-dichlorobenzene (1:1) as a co-solvent over 72 h at 115 °C in Schlenk tubes, in the presence of acetic acid (6 M, 10 vol%) as an acidic catalyst. The molecular structures of the hydroxyl-functionalized COFs were verified using FTIR and a solid state ^13^C NMR spectroscopy. According to the PXRD, BET, TGA, and XPS measurements, hydroxyl-functionalized COFs featured high BET-specific surface areas up to 1089 m^2^ g^−1^, excellent crystallinity, and a superior thermal stability up to 60.44% char yield. In addition, we evaluated the applicability of our hydroxyl-functionalized COFs for a supercapacitor application. The resultant hydroxyl-functionalized COFs demonstrated an outstanding electrochemical efficiency (271 F g^−^^1^ at a current density of 0.5 A g^−^^1^) because of the presence of redox-active 2,3-dihydroxynaphthalene and 2,6-dihydroxynaphthalene in their COF skeletons. Furthermore, we believe that these novel hydroxyl-functionalized COFs might be useful in a variety of applications, including energy storage technologies.

## Data Availability

Data supporting the findings of this study are available on request by the corresponding author.

## Supplementary Materials

The following supporting information can be downloaded at: https://www.mdpi.com/article/10.3390/polym14163428/s1, Table S1: PXRD data and BET parameters of the synthesized TAPT-2,3-NA(OH)_2_, TAPT-2,6-NA(OH)_2_ COFs; Table S2: Fractional atomic coordinates for the unit cell of TAPT-2,3-NA(OH)_2_ COF with A–A stacking; Table S3: Fractional atomic coordinates for the unit cell of TAPT-2,6-NA(OH)_2_ COF with A–A stacking; Table S4: XPS fitting positions of TAPT-2,3-NA(OH)_2_ and TAPT-2,6-NA(OH)_2_ COF; Table S5: XPS fitting ratio of TAPT-2,3-NA(OH)_2_ and TAPT-2,6-NA(OH)_2_ COF; Table S6: Values of T*_d_*_10%_, and Char yield of TAPT-2,3-NA(OH)_2_ and TAPT-2,6-NA(OH)_2_ COFs; Table S7: Comparison between the specific surface area/specific capacitance of TAPT-2,3-NA(OH)_2_ and (b) TAPT-2,3-NA(OH)_2_ COFs with those of previously reported COFs for supercapacitor application; Scheme S1: Synthesis of 1,3,5-tris-(4-aminophenyl)triazine (TPT-3NH2); Scheme S2: Synthesis of 2,3-dihydroxynaphthalene-1,4-dicarbaldehyde (2,3-NADC); Scheme S3: Synthesis of 2,6-dihydroxynaphthalene-1,5-dicarbaldehyde (2,6-NADC); Scheme S4: Synthesis of TAPT-2,3-NA(OH)_2_ COF; Scheme S5: Synthesis of TAPT-2,6-NA(OH)_2_ COF; Figure S1: FT-IR spectrum of 1,3,5-tris-(4-aminophenyl)triazine; Figure S2: ^1^H-NMR of 1,3,5-tris-(4-aminophenyl)triazine; Figure S3: ^13^C-NMR of 1,3,5-tris-(4-aminophenyl)triazine; Figure S4: FT-IR spectrum of 2,3-dihydroxynaphthalene-1,4-dicarbaldehyde; Figure S5: ^1^H-NMR of 2,3-dihydroxynaphthalene-1,4-dicarbaldehyde; Figure S6: ^13^C-NMR of 2,3-dihydroxynaphthalene-1,4-dicarbaldehyde; Figure S7: FT-IR spectrum of 2,6-dihydroxynaphthalene-1,5-dicarbaldehyde; Figure S8: ^1^H-NMR of 2,6-Dihydroxynaphthalene-1,5-dicarbaldehyde; Figure S9: ^13^C-NMR of 2,6-Dihydroxynaphthalene-1,5-dicarbaldehyde; Figure S10: Experimental and simulated PXRD patterns of TAPT-2,3-NA(OH)_2_ COF; Figure S11: Experimental and simulated PXRD patterns of TAPT-2,6-NA(OH)_2_ COF; Figure S12: Crystalline structures for the TAPT-2,3-NA(OH)_2_ and TAPT-2,6-NA(OH)_2_ COFs with completely eclipsed AA-stacking models; 3D-view of the simulated structures of the COFs along (**a**,**c**) the c axis and (**b**,**d**) the a axis; Figure S13: FTIR spectrum of 2,3-NADC, TAPT-3NH_2_, and TAPT-2,3-NA(OH)_2_ COFs; Figure S14: FTIR spectrum of 2,6-NADC, TAPT-3NH_2_, and TAPT-2,6-NA(OH)_2_ COFs; Figure S15: XPS spectra of TAPT-2,3-NA(OH) and TAPT-2,6-NA(OH)_2_ COFs; Figure S16: XPS spectra of the (**a**,**d**) C1s, (**b**,**e**) N1s and (**c**,**f**) O1s peaks for (**a**–**c**) TAPT-2,3-NA(OH) and (**d**–**f**) TAPT-2,6-NA(OH)_2_ COFs; Figure S17: TEM images of the TAPT-2,3-NA(OH)_2_ COF recorded at various magnifications: (**a**) 1 µm, (**b**) 0.5 µm; Figure S18: TEM images of the TAPT-2,6-NA(OH)_2_ COF recorded at various magnifications: (**a**) 200 nm, (**b**) 100 nm; Figure S19: FE-SEM images of the (**a**,**b**) TAPT-2,3-NA(OH)_2_ COF, (**c**,**d**) TAPT-2,6-NA(OH)_2_ COF, recorded at various magnifications: (a, c) 1 μm and (b, d) 100 nm; Figure S20: Comparison CV curves of the TAPT-2,3-NA(OH)_2_ and TAPT-2,6-NA(OH)_2_ COFs, measured at a scan rate (5 mV s^−1^). Figure S21: CV curves of the (**a**) TAPT-2,3-NA(OH)_2_ and (**b**) TAPT-2,6-NA(OH)_2_ COFs, measured at a scan rate (5 mV s^−1^). Redox transformation of the (**c**) TAPT-2,3-NA(OH)_2_ COF, (**d**) TAPT-2,6-NA(OH)_2_ COF. Figure S22: GCD curve of TAPT-2,3-NA(OH)_2_ and TAPT-2,6-NA(OH)_2_ COFs, measured at a current density (0.5 A g^−1^); Figure S23: Electrochemical impedance spectroscopy (Nyquist plots) of TAPT-2,3-NA(OH)_2_ and TAPT-2,6-NA(OH)_2_ COFs. Figure S24: FTIR spectra of TAPT-2,3-NA(OH)_2_ and TAPT-2,6-NA(OH)_2_ COFs as-synthesized and after soaking 2 days in KOH (1 M).
