# Supplementary material for: Hydroxyl-Functionalized Covalent Organic Frameworks as High-Performance Supercapacitors
Source: Polymers (Basel). 2022 Aug 22;14(16):3428. doi: 10.3390/polym14163428 (PMC9413307; doi:10.3390/polym14163428)
Supplement: Supplementary file 1 [file polymers-14-03428-s001.zip › polymers-1861996-supplementary.pdf]

Electronic Supplementary Information (ESI)

Hydroxyl-Functionalized Covalent Organic Frameworks as High-Performance Supercapacitors

Tzu-Ling Yang [1], Jhu-You Chen [1], Shiao-Wei Kuo [1], Chen-Tsyr Lo [2,*] and Ahmed F. M. EL-Mahdy [1,3,*]

[1] Department of Materials and Optoelectronic Science, National Sun Yat-Sen University, Kaohsiung 80424, Taiwan

[2] Department of Material Science and Engineering, National Taiwan University of Science and Technology, Taipei 10617, Taiwan

[3] Chemistry Department, Faculty of Science, Assiut University, Assiut 71516, Egypt

* Correspondence: lochentsyr@mail.ntust.edu.tw (C.-T.L.); ahmedelmahdy@mail.nsysu.edu.tw (A.F.M.E.-M.); Tel.: +886-2-2737-6527 (C.-T.L.); +886-7-5252-000 (ext. 4002) (A.F.M.E.-M.)

S1. Materials

2,3-Dihydroxynaphthalene, 2,6-dihydroxynaphthalene, 4-bromoaniline, potassium carbonate, and formamidine were received from Acros. Acetic anhydride, HCl, and triflic acid were ordered from Alfa Aesar. Dioxane, chloroform, *n*-butanol and *o*-dichlorobenzene, acetic acid, DMF, THF, and acetone were obtained from J. T. Baker. Raw chemical materials and solvents were purchased from commercial resources and then used as they were.

S2. Characterization

A Bruker Tensor 27 FTIR spectrophotometer was used to measure FTIR spectra via KBr disk method; every scan was recorded by a 4 cm^{-1} resolution range. An INOVA 500 instrument was employed for recording ^{13}C NMR and ^{1}H spectra by DMSO-d_6 and $CDCl_3$ as solvents and tetramethylesilane (TMS) as exterior standard. Chemical shifts were recorded by a part per million (ppm) scale. A Bruker Avance 400 NMR spectrometer in addition to a Bruker magic-angle-spinning (MAS) sensor were applied for recording the solid state nuclear magnetic resonance (SSNMR) spectra. ^{13}C NMR spectral information was registered at 75.5 MHz via cross-polarization with MAS (CPMAS). The CP contact time was 2 ms, while data acquiring ^{1}H decoupling was applied by a decoupling frequency of 32 KHz and an MAS sample spinning rate of 10 kHz. Nitrogen adsorption–desorption measurements were carried out using a BelSorp max instrument. Before measuring the gas adsorption, the as-prepared samples (50 mg) were washed with anhydrous tetrahydrofurane for 24 hours using Soxhlet extraction. The solvent was filtered, and the samples were activated for 10 hours under pressure at 150 °C. The samples were then used for gas adsorption–desorption measurements at 77 K from 0 to 1 atm. Their specific surface areas were calculated using the Brunauer–Emmett–Teller (BET) methodology. The pore distributions were calculated from the sorption data using the quenched solid state density functional theory. TA Q-50 analyzer under a flow of N_2, TGA data were examined. In a well-sealed Pt cell and under a nitrogen flow rate of 50 mL min^{-1}; samples were exposed to increasing heat from 40 to 800 °C, at heating ramp 20 °C min^{-1}. A JEOL JSM-7610F scanning electron microscope was used for visualization of FE-SEM, and samples were exposed to Pt sputtering for 100 s for handling observation. A JEOL-2100 scanning electron microscope, operated at 200 kV, was used for TEM images.

S3. Synthetic Procedure of Monomers

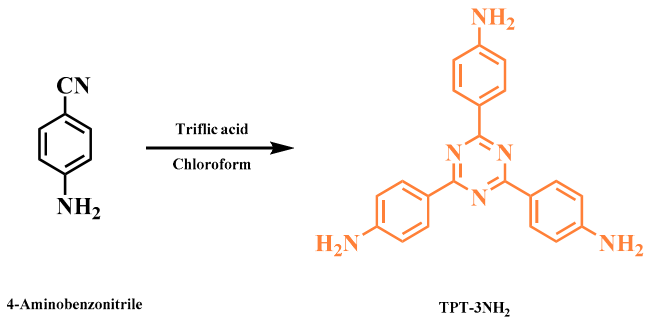

Scheme S1. Synthesis of 1,3,5-tris-(4-aminophenyl)triazine (TPT-3NH_2).

1,3,5-Tris-(4-aminophenyl)triazine (TPT-3NH_2) : A solution of 4-aminobenzonitrile (3.09 g, 26.1 mmol) in $CHCl_3$ (40 mL) was placed in a round-bottom flask at 0 °C. TfOH (8.00 mL, 30.4 mmol) was added dropwise over 15 minutes while maintaining the temperature at 0 °C. The mixture was stirred at room temperature for 24 h under an N_2 atmosphere. Ice-cooled distilled H_2O (50 mL) was added and then the mixture was neutralized by adding 2 M NH_4OH until the pH exceeded 7. Initially, upon increasing pH, the orange precipitate dissolved to give a bright-orange solution. A further increase in pH provided a pale-yellow precipitate, which was filtered off and then washed three times with distilled H_2O and MeOH. The solvent was evaporated slowly through using vacuum

distillation. The residue was dried in a vacuum oven to provide a pale-yellow powder (2.81 g, 91%). ^{1}H NMR (DMSO-d_6, 25°C, 600 MHz) δ (ppm): 8.36 (d, *J* = 10.2 Hz, 6H), 6.70 (d, *J* = 10.2 Hz, 6H), 5.89 (s, 6H). ^{13}C NMR (DMSO-d_6, 25 °C, 125 MHz) δ (ppm): 170.45, 153.77, 130.82, 123.56, 113.69.

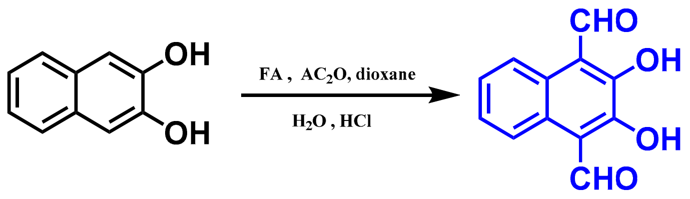

2,3-Dihydroxynaphthalene

2,3-NADC

Scheme S2. Synthesis of 2,3-dihydroxynaphthalene-1,4-dicarbaldehyde (2,3-NADC).

2,3-Dihydroxynaphthalene-1,4-dicarbaldehyde (2,3-NADC): Formamidine acetate (1.56 g, 14.98 mmol) and dioxane (30 mL) were added into a 100-mL two-neck round-bottom flask and heated under reflux. Acetic anhydride (3mL) was added when the target temperature reached 95 °C and was then stirred for 30 minutes. 2,3-Dihydroxynaphthalene (300 mg, 1.87 mmol) was added once all formamidine acetate was dissolved, and kept for two days. After cooling for minutes, dioxane was evaporated at 50 °C, was added H_2O (45mL), and heated at 65 °C for 2 hours. Thereafter, we added HCl (1 M, 40 mL) and kept heating at 65 °C for 18 hours. The solid was filtered and washed several times with hexane. 2,3-Dihydroxynaphthalene-1,4-dicarbaldehyde was obtained after purifying by using column chromatography with THF/hexane as eluent (303 mg, 75% yield). FTIR: 3380, 2890-2965, and 1674 cm^{-1}. ^{1}H NMR (DMSO-d_6, 25 °C, 500 MHz) δ (ppm): 10.68 (s, 2H), 8.29 (d, J = 13.0 Hz, 2H), and 7.38 (d, *J* = 13.0 Hz, 2H). ^{13}C NMR (DMSO-d_6, 25 °C, 125 MHz) δ (ppm): 195.31, 155.0, 127.13, 125.77, 123.21, and 117.76.

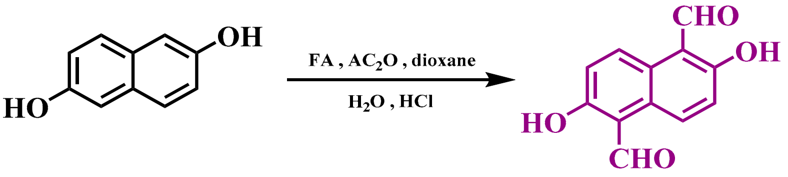

2,6-Dihydroxynaphthalene

2,3-NADC

Scheme S3. Synthesis of 2,6-dihydroxynaphthalene-1,5-dicarbaldehyde (2,6-NADC).

2,6-dihydroxynaphthalene-1,5-dicarbaldehyde (2,6-NADC): Formamidine acetate (1.56 g, 14.98 mmol) and dioxane (45 mL) were heated under reflux in a 100-mL two-neck round-bottom flask. Acetic anhydride (3mL) was added when the target temperature reached 95 °C and stirred for thirty minutes. 2,6-Dihydroxynaphthalene (300 mg, 1.87 mmol) was added once all formamidine acetate was dissolved and kept for two days. After cooling for minutes, dioxane was evaporated at 50 °C, was added H_2O (45mL) and was heated at 65 °C for 2 hours. Thereafter, we added HCl (1 M, 45 mL) and kept heating at 65 °C for 18 hours. The solid was filtered and washed several times with hexane. 2,6-Dihydroxynaphthalene-1,5-dicarbaldehyde was obtained after purifying by using column chromatography with THF/hexane as eluent (291 mg, 72% yield). FTIR: 3445, 2852-2996, 3058, and 1640 cm^{-1}. ^{1}H NMR (DMSO-d_6, 25 °C, 500 MHz) δ (ppm): 10.75 (s, 2H), 9.14 (d, J = 12 Hz, 2H), and 7.32 (d, *J* = 12 Hz, 2H). ^{13}C NMR (DMSO-d_6, 25 °C, 125 MHz) δ (ppm): 193.81, 162.35, 133.05, 126.43, 121.93, and 113.92.

S4. Synthetic Procedure of COFs

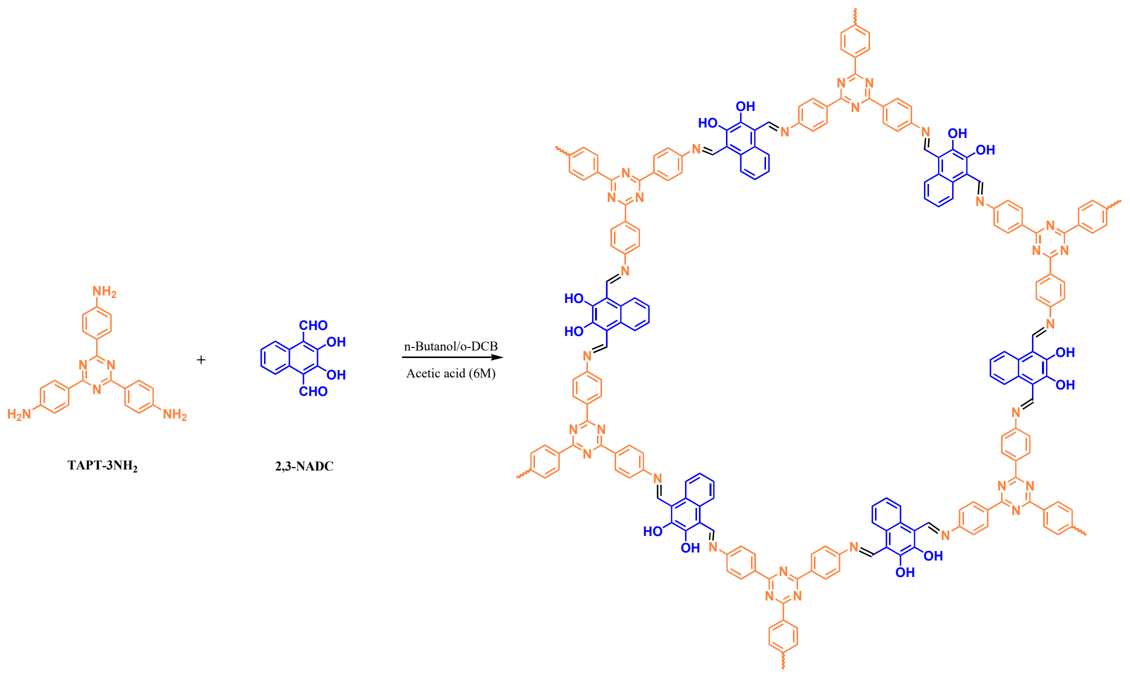

TAPT-2,3-NA(OH)$_2$

Scheme S4. Synthesis of TAPT-2,3-NA(OH)$_2$ COF.

TAPT-2,3-NA(OH)$_2$ COF: Three freeze/pump/thaw cycles were used to degas a solution of TAPT-3NH$_2$ (70.0 mg, 0.196 mmol) and 2,3-NADC (64.1 mg, 0.296 mmol) in n-butanol (3.5 mL) and o-dichlorobenzene (3.5 mL) with acetic acid (6 M, 0.7 mL) in a 25-mL Schlenk storage tube. The tube was then flame-sealed and heated at 120 °C for three days. After the tube had cooled to room temperature, the precipitate was filtered and washed (once with DMF, three times each with THF and acetone). The solid was vacuum-dried overnight at 120 °C to provide the TAPT-2,3-NA(OH)$_2$ COF as a red-brown powder (172 mg, 84% yield).

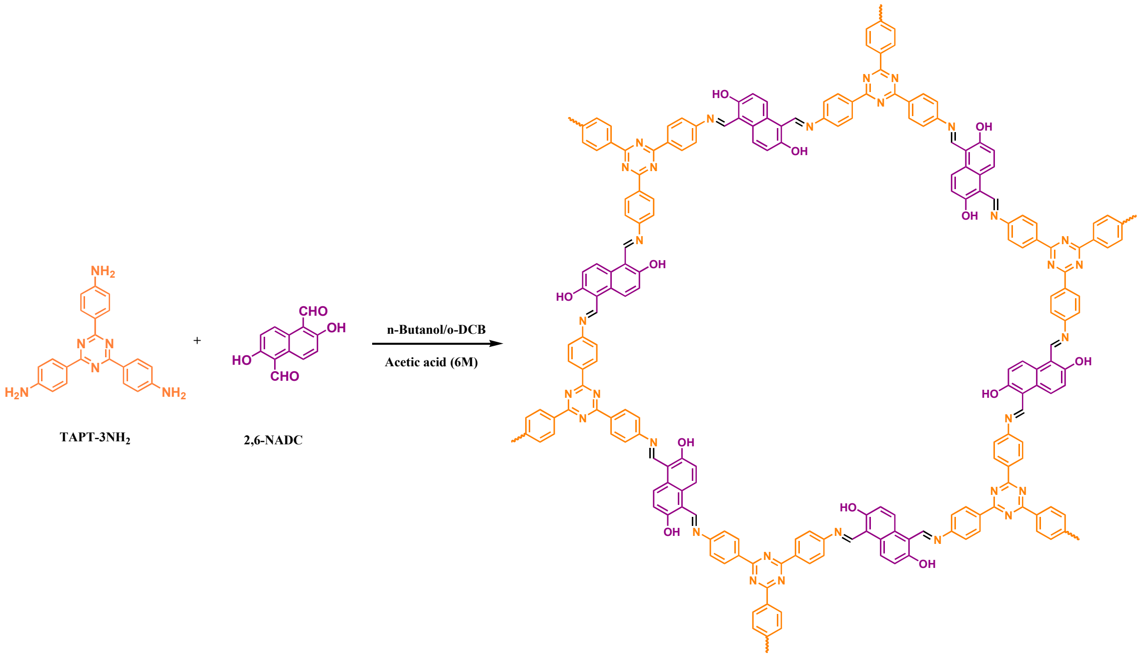

TAPT-2,6-NA(OH)2

Scheme S5. Synthesis of TAPT-2,6-NA(OH)2 COF.

TAPT-2,6-NA(OH)2 COF: Three freeze/pump/thaw cycles were used to degas a solution of TAPT-3NH2 (70.0 mg, 0.196 mmol) and 2,6-NADC (64.6 mg, 0.299 mmol) in n-butanol (3.5 mL) and o-dichlorobenzene (3.5 mL) with acetic acid (6 M, 0.7 mL) in a 25-mL Schlenk storage tube. The tube was flame-sealed and heated at 120 °C for three days. After the tube had cooled to room temperature, the precipitate was filtered and washed (once with DMF, three times each with THF and acetone). The solid was vacuum-dried overnight at 120 °C to get the TAPT-2,6-NA(OH)2 COF as a red-brown powder (176 mg, 86% yield).

S5. FTIR and NMR Spectral Profiles of Monomers

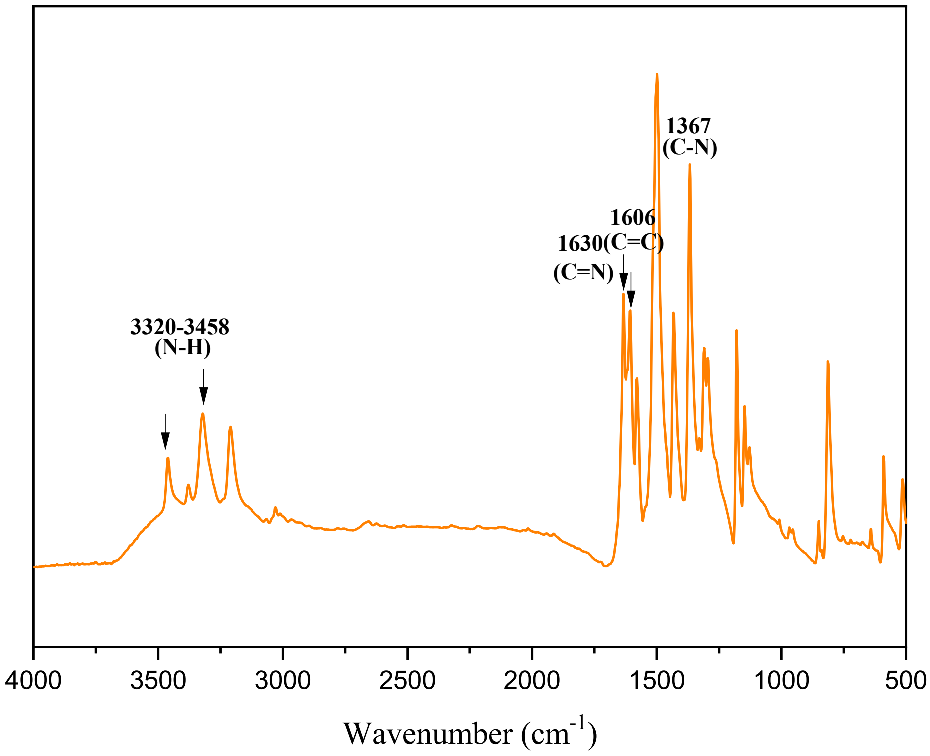

Figure S1. FT-IR spectrum of 1,3,5-tris-(4-aminophenyl)triazine.

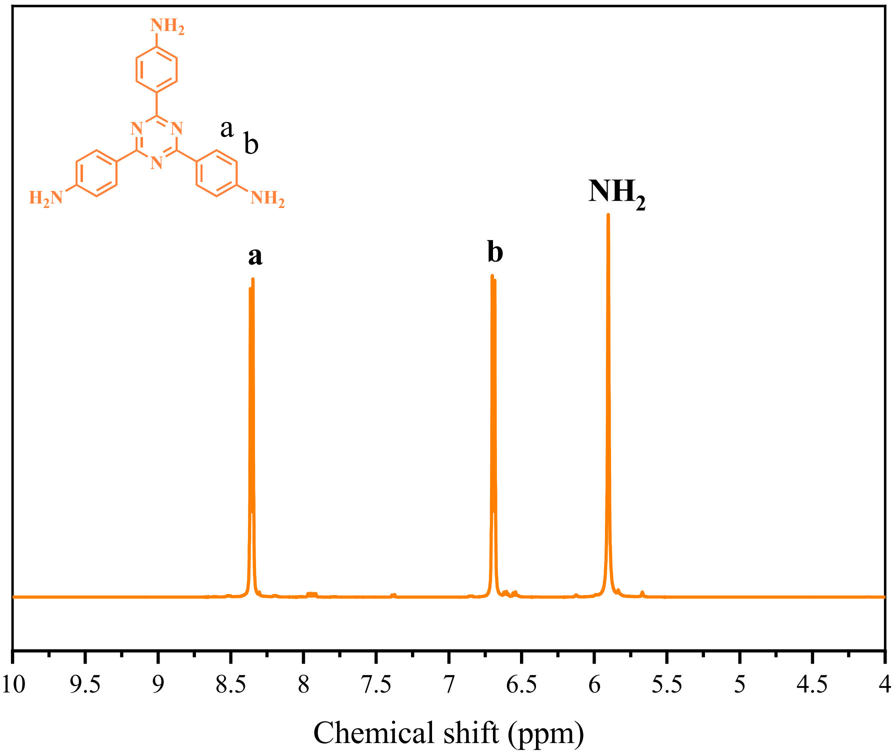

Figure S2. ^{1}H-NMR of 1,3,5-tris-(4-aminophenyl)triazine.

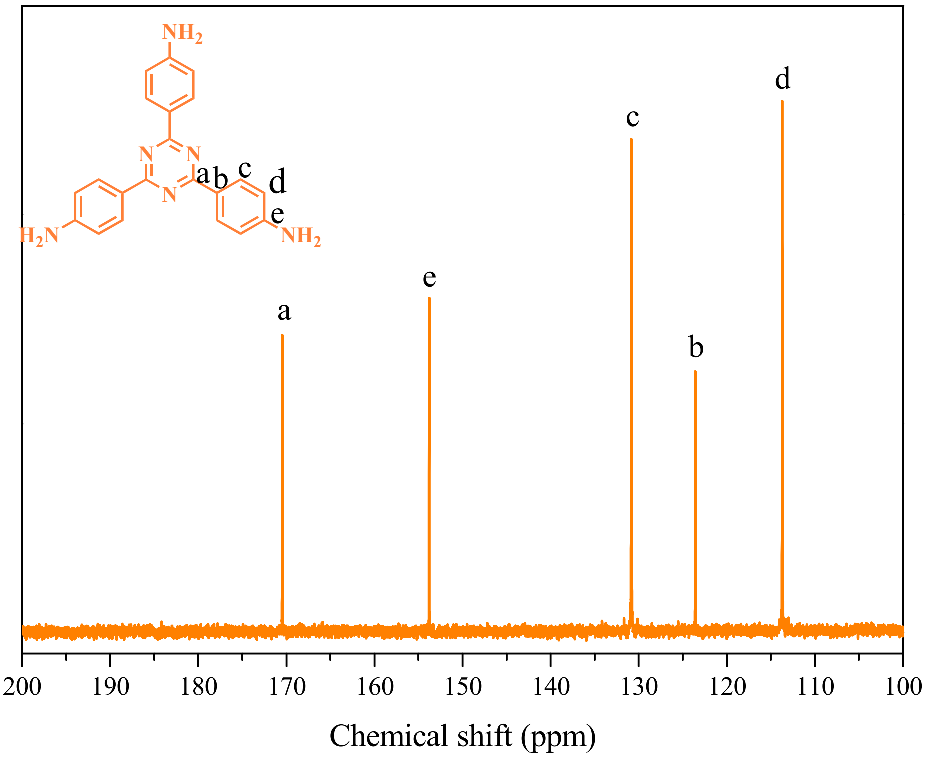

Figure S3. ^{13}C-NMR of 1,3,5-tris-(4-aminophenyl)triazine.

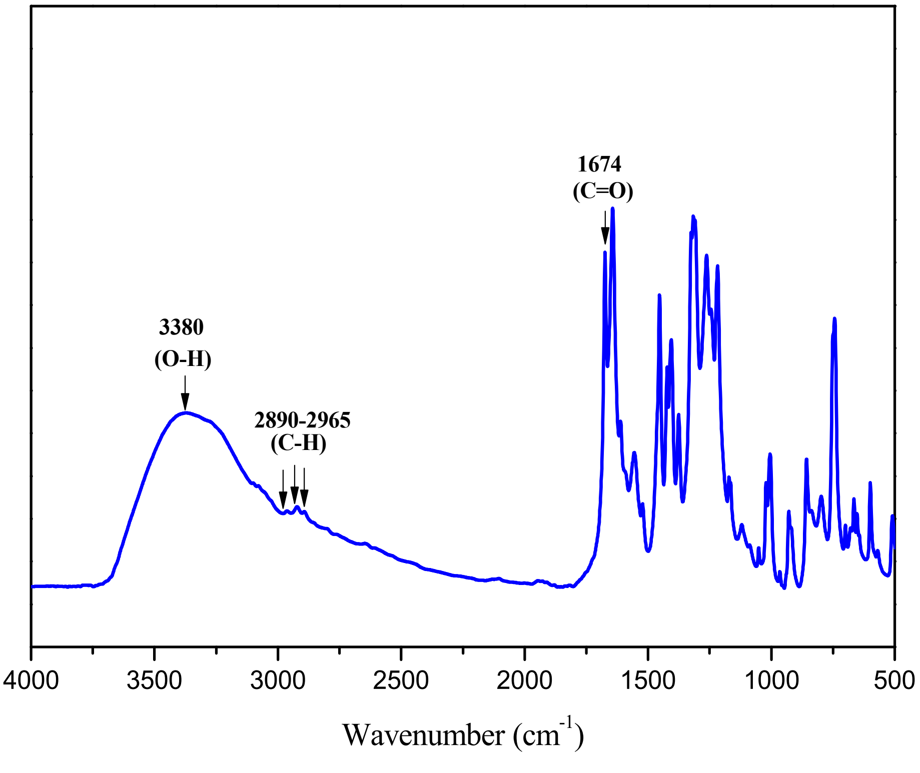

Figure S4. FT-IR spectrum of 2,3-dihydroxynaphthalene-1,4-dicarbaldehyde.

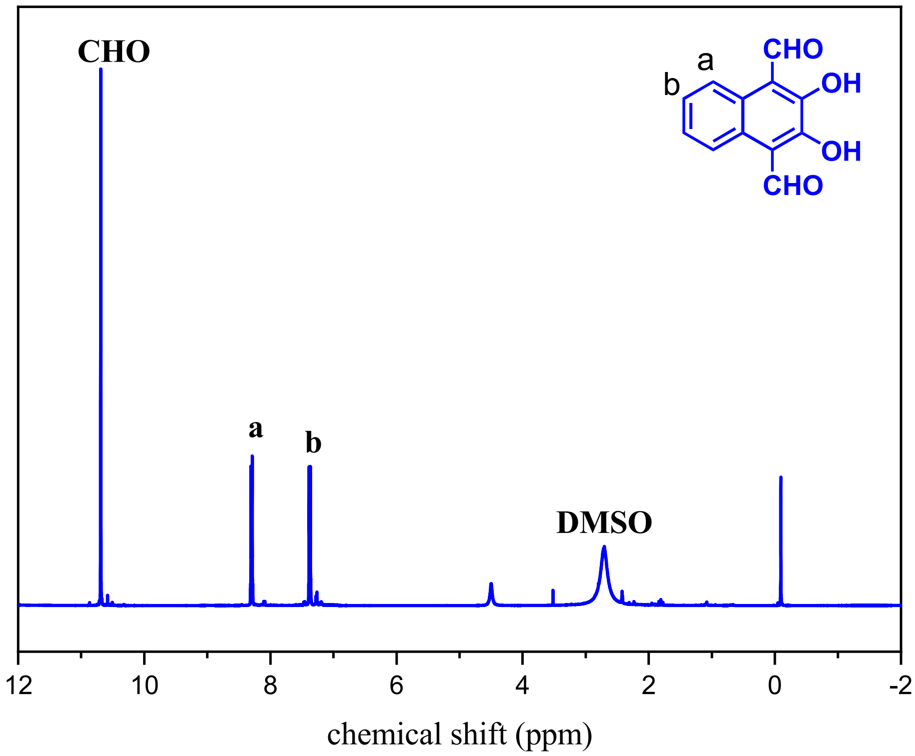

Figure S5. ^{1}H-NMR of 2,3-dihydroxynaphthalene-1,4-dicarbaldehyde.

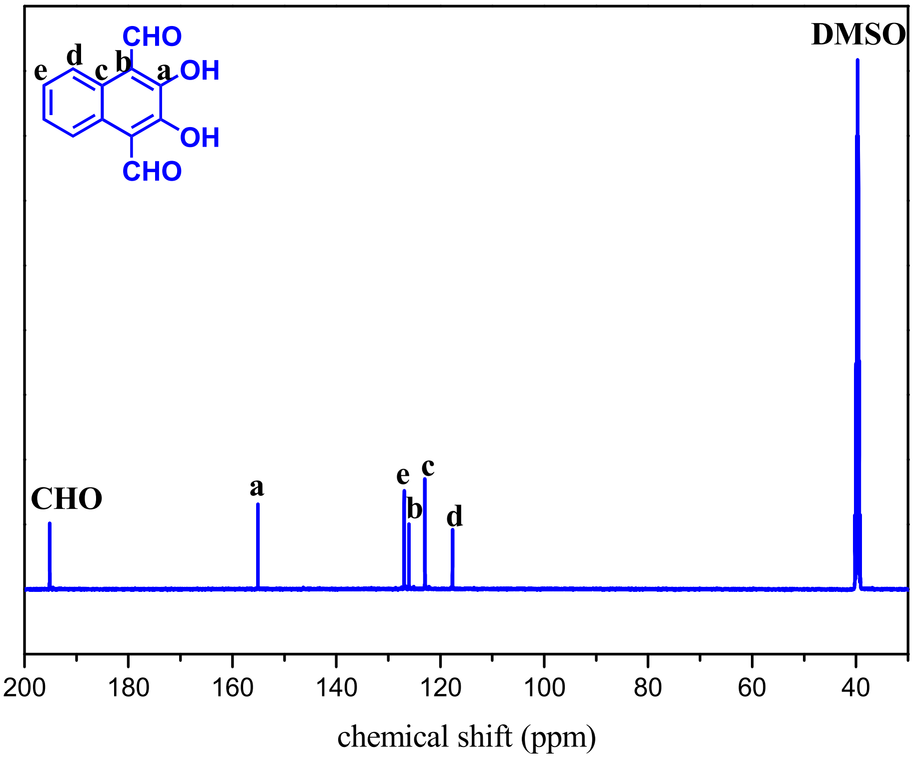

Figure S6. ^{13}C-NMR of 2,3-dihydroxynaphthalene-1,4-dicarbaldehyde.

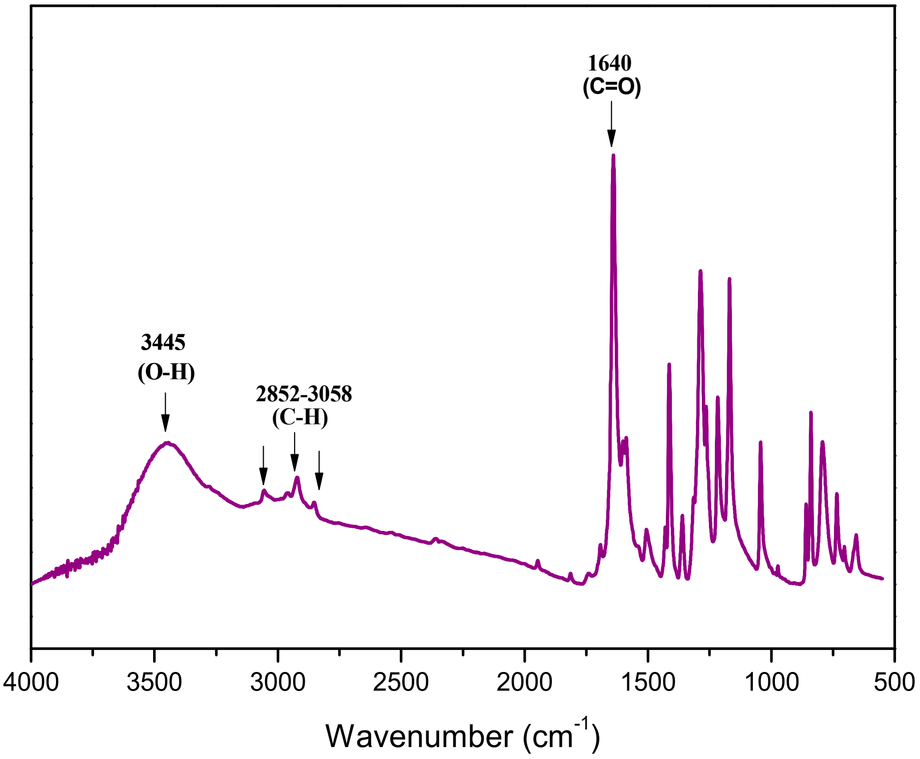

Figure S7. FT-IR spectrum of 2,6-dihydroxynaphthalene-1,5-dicarbaldehyde.

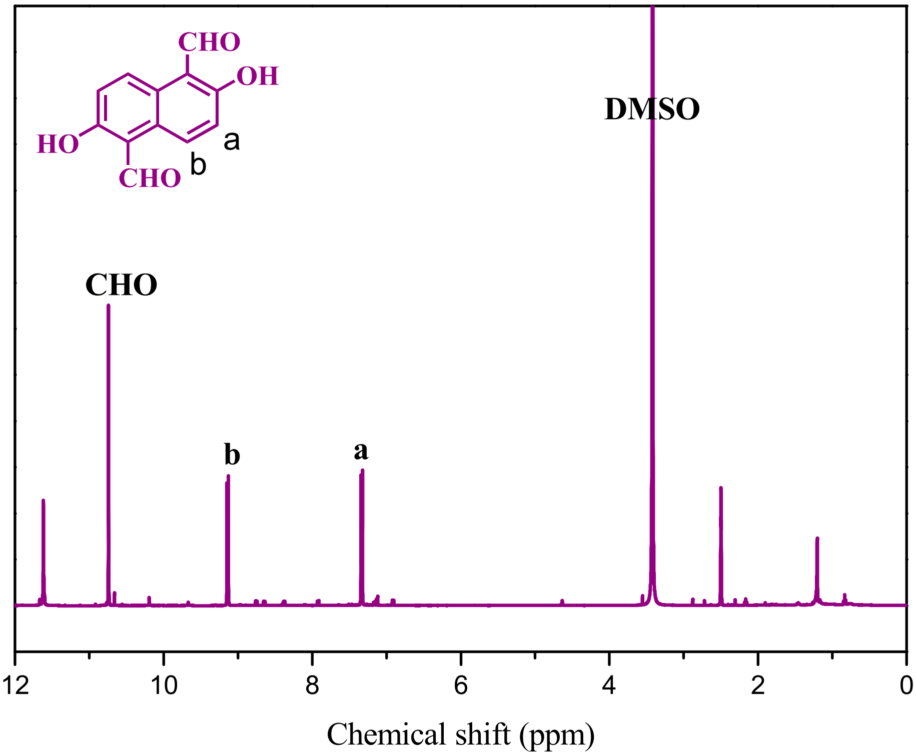

Figure S8. ^{1}H-NMR of 2,6-dihydroxynaphthalene-1,5-dicarbaldehyde.

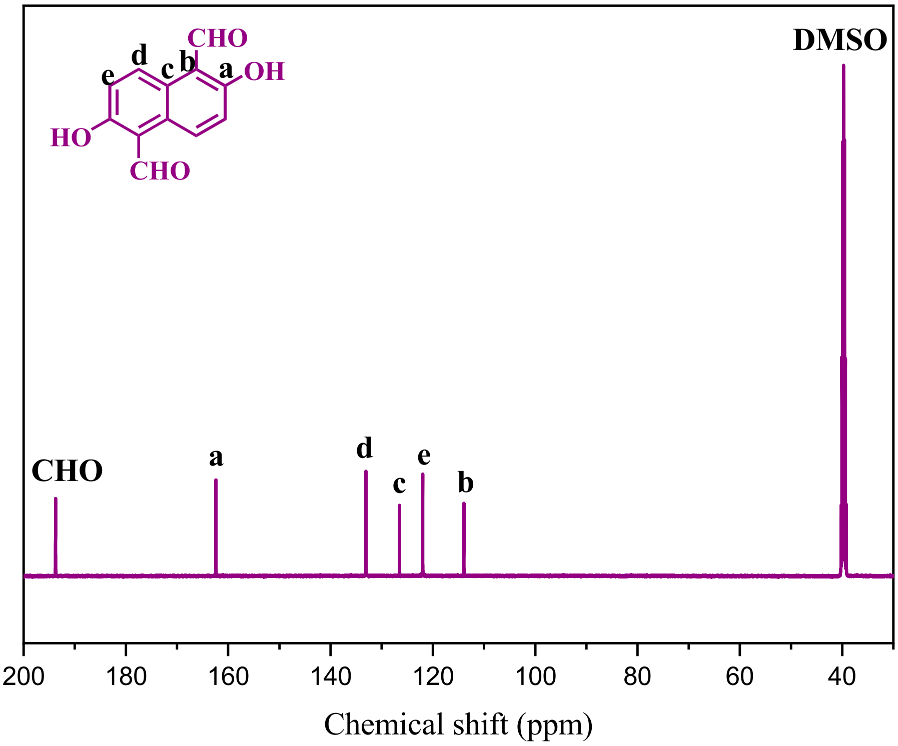

Figure S9. ^{13}C-NMR of 2,6-dihydroxynaphthalene-1,5-dicarbaldehyde.

S6. PXRD data and BET parameters

Table S1. PXRD data and BET parameters of the synthesized TAPT-2,3-NA(OH)$_2$, TAPT-2,6-NA(OH)$_2$ COFs.

COFs	S_{BET} (m^2 g^{-1})	d_{110} (nm)	Pore size (nm)	Interlayer distance (Å)	Pore Volume (cm^3 g^{-1})
**TAPT-2,3-NA(OH)$_2$**	429	3.76	3.04	3.96	0.17
**TAPT-2,6-NA(OH)$_2$**	1089	3.78	3.29	3.94	0.22

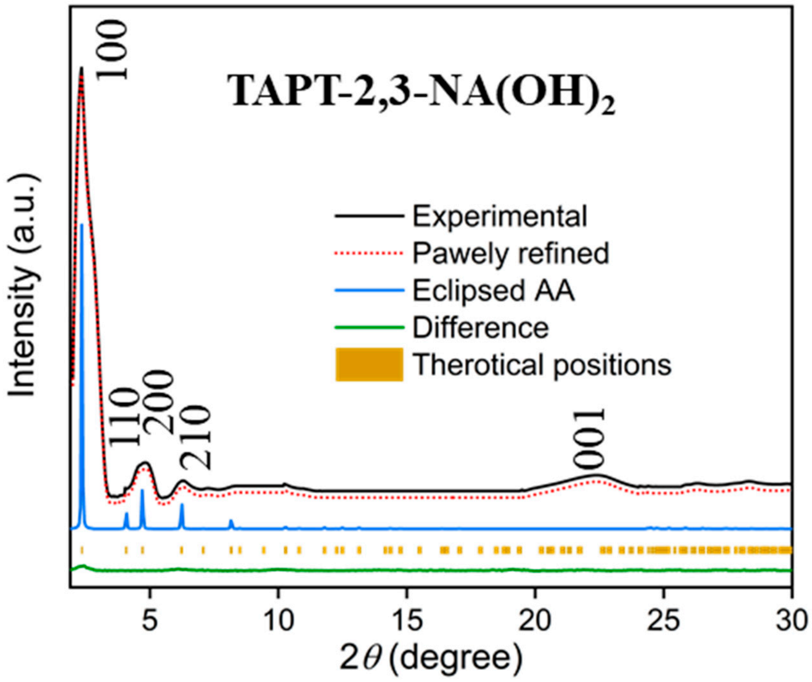

Figure S10. Experimental and simulated PXRD patterns of TAPT-2,3-NA(OH)$_2$ COF.

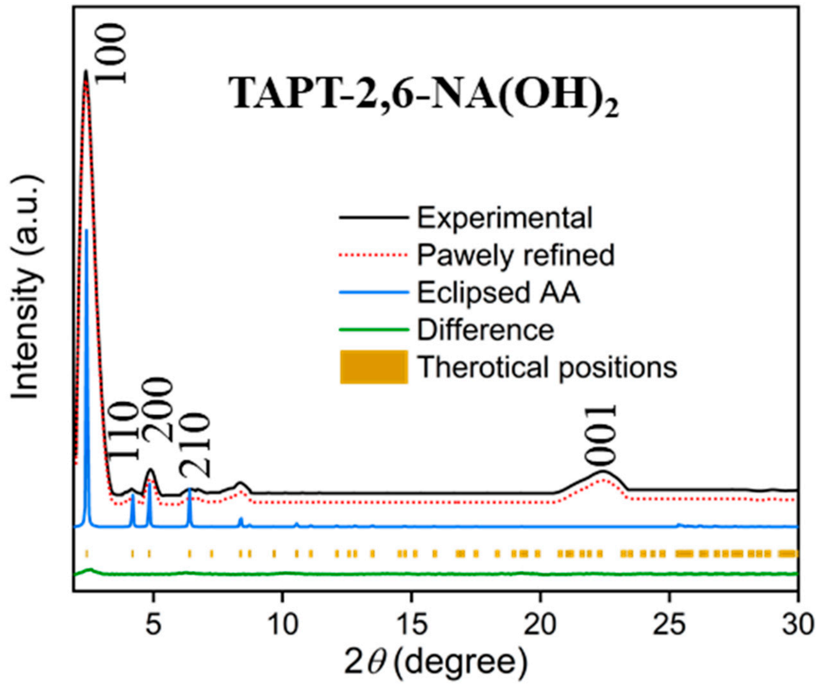

Figure S11. Experimental and simulated PXRD patterns of TAPT-2,6-NA(OH)$_2$ COF.

S7. Structural Modeling and Fractional Atomic Coordinates for COF Structures

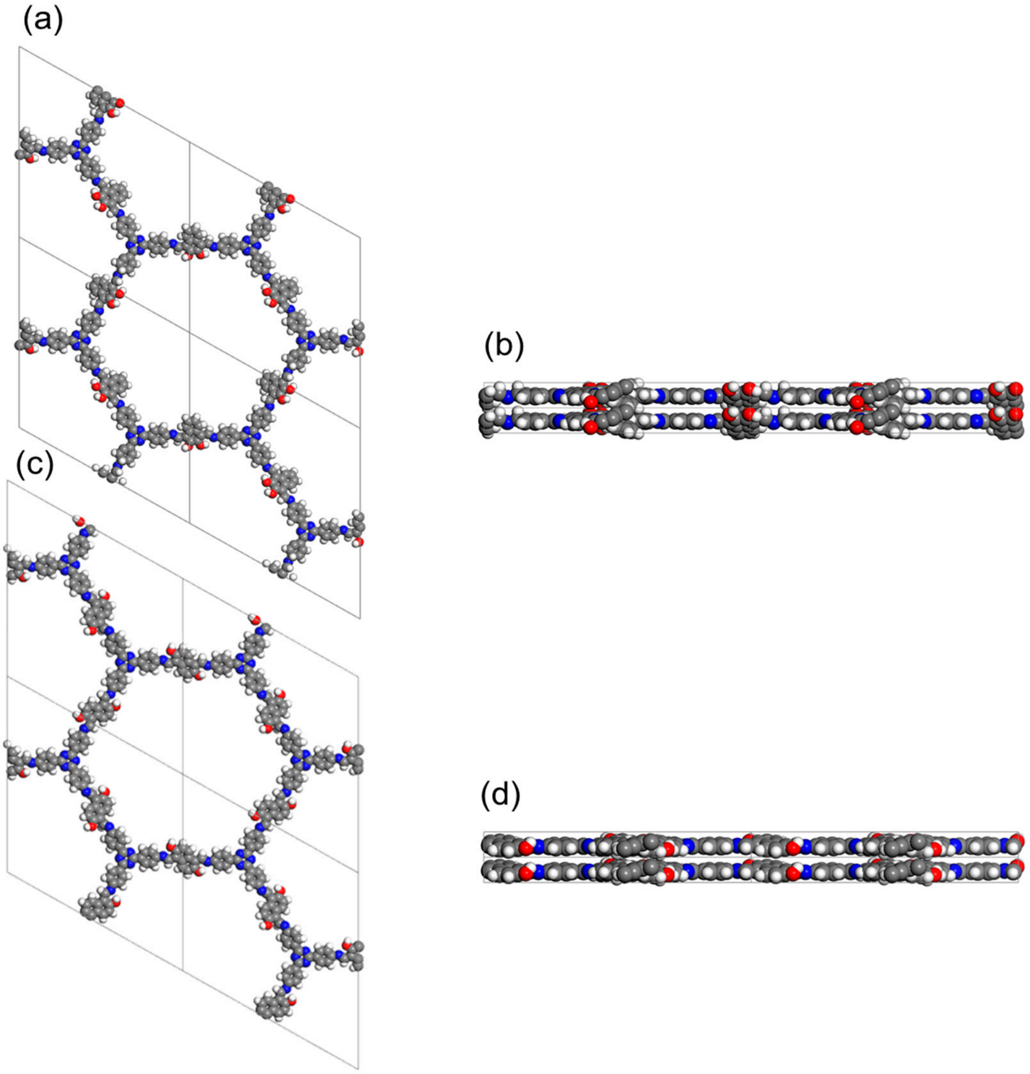

Figure S12. Crystalline structures for the TAPT-2,3-NA(OH)2 and TAPT-2,6-NA(OH)2 COFs with completely eclipsed AA-stacking models; 3D-view of the simulated structures of the COFs along (a, c) the c axis and (b, d) the a axis.

Table S2. Fractional atomic coordinates for the unit cell of TAPT-2,3-NA(OH)2 COF with A–A stacking.

Sample name: TAPT-2,3-NA(OH)2 COF							
Space group: P 1							
a = 43.4 Å, b = 43.2 Å, c = 3.649 Å, $\alpha = \beta = 90°$, $\gamma = 120°$							
R_{wp} = 7.45%, R_p = 456%							
**Atom**	***x/a***	***y/b***	***z/c***	**Atom**	***x/a***	***y/b***	***z/c***
C1	0.48504	0.95082	0.32530	C73	0.55599	0.41496	0.50346
C2	0.49479	0.92419	0.44905	C74	0.54037	0.44444	0.48084
C3	0.53090	0.94337	0.58646	C75	0.55748	0.48121	0.35328
C4	0.43837	0.96020	0.06296	C76	0.54025	0.50440	0.33938
C5	0.45080	0.93757	0.17442	C77	0.50359	0.49392	0.45657
C6	0.46543	0.87897	0.47164	C78	0.48668	0.45704	0.58504
O7	0.54357	0.92174	0.72926	C79	0.50408	0.43402	0.59447
N8	0.46800	0.84796	0.45399	C80	0.59279	0.49598	0.23317
C9	0.44291	0.80550	0.46279	C81	0.61185	0.53087	0.10380

C10	0.40428	0.78395	0.47854	C82	0.59619	0.55204	0.08311
C11	0.38303	0.74365	0.47944	C83	0.56117	0.53911	0.19486
C12	0.39712	0.71907	0.46549	C84	0.48765	0.52326	0.48068
C13	0.43566	0.74114	0.45023	O85	0.48478	0.39918	0.73529
C14	0.45674	0.78105	0.44814	O86	0.45202	0.44195	0.72583
C15	0.37371	0.67318	0.46618	N87	0.45415	0.52050	0.45495
N16	0.33578	0.65279	0.46578	N88	0.57783	0.08780	0.41797
C17	0.31516	0.61472	0.46602	C89	0.57051	0.05515	0.57732
N18	0.33557	0.59716	0.46647	C90	0.54464	0.01042	0.48246
C19	0.37342	0.61441	0.46687	C91	0.50840	-0.00876	0.34450
N20	0.39094	0.65243	0.46679	C92	0.55441	-0.01624	0.59907
C21	0.39587	0.59103	0.46679	C93	0.49406	0.01235	0.22716
C22	0.26941	0.59149	0.46630	C94	0.45957	-0.00295	0.09142
C23	0.24514	0.60589	0.44716	O95	0.58876	-0.00312	0.74222
C24	0.20499	0.58481	0.44768	C96	-3.41184	0.15701	1.50413
C25	0.18322	0.54603	0.46854	H97	0.41194	0.94892	-0.05266
C26	0.20737	0.53190	0.48850	H98	0.43316	0.90936	0.13158
C27	0.24713	0.55282	0.48645	H99	0.43845	0.87340	0.50637
C28	0.37957	0.55236	0.44476	H100	0.56848	0.93603	0.83443
C29	0.39846	0.53140	0.44268	H101	0.38972	0.79819	0.48959
C30	0.43665	0.54546	0.46468	H102	0.35439	0.73147	0.49194
C31	0.45354	0.58423	0.48750	H103	0.45048	0.72713	0.43802
C32	0.43444	0.60535	0.48788	H104	0.48539	0.79336	0.43397
N33	0.14085	0.52110	0.47781	H105	0.25747	0.63467	0.43049
C34	0.11010	0.52389	0.45467	H106	0.19102	0.59962	0.43228
C35	0.06496	0.49447	0.47794	H107	0.19498	0.50314	0.50705
C36	0.03856	0.50468	0.59629	H108	0.26087	0.53774	0.50302
C37	0.04529	0.45764	0.34889	H109	0.35084	0.53735	0.42652
C38	0.05203	0.53936	0.74091	H110	0.38219	0.50265	0.42240
C39	0.02977	0.55219	0.85156	H111	0.48225	0.59899	0.50451
O40	0.06496	0.44278	0.20491	H112	0.45079	0.63413	0.50611
C41	0.99821	0.48132	0.58197	H113	0.11581	0.55098	0.41255
C42	0.97875	0.44445	0.45567	H114	0.08015	0.55706	0.78039
C43	1.00519	0.43425	0.34620	H115	0.04124	0.57895	0.96229
C44	0.99306	0.53095	0.82932	H116	0.09164	0.45877	0.22410
C45	0.97752	0.49606	0.70008	H117	0.97634	0.54144	0.92068
C46	0.93357	0.41564	0.41805	H118	0.94878	0.48039	0.69545
O47	0.99180	0.39934	0.21712	H119	0.92795	0.39057	0.29637
N48	0.90273	0.41710	0.49884	H120	0.97330	0.38109	0.38866
C49	0.86038	0.39243	0.47701	H121	0.84844	0.43407	0.60775
C50	0.83616	0.40589	0.54521	H122	0.78261	0.39955	0.59500
C51	0.79640	0.38502	0.53810	H123	0.78626	0.30487	0.34396
C52	0.77419	0.34693	0.46799	H124	0.85273	0.34001	0.34885
C53	0.79854	0.33315	0.40212	H125	0.60350	0.12700	0.85228
C54	0.83868	0.35428	0.40402	H126	0.65733	0.13925	0.38660
C55	0.72846	0.32355	0.46647	H127	0.69095	0.20460	0.35776
N56	0.70776	0.34078	0.46986	H128	0.69186	0.39968	0.38442
C57	0.66987	0.32320	0.47168	H129	0.66051	0.43430	0.37574
N58	0.65257	0.28512	0.47283	H130	0.56127	0.33892	0.55154
C59	0.67022	0.26477	0.46814	H131	0.59275	0.30373	0.55488
N60	0.70810	0.28554	0.46365	H132	0.53471	0.38874	0.58259

C61	0.64727	0.34643	0.47065	H133	0.60600	0.48036	0.23727
C62	0.64832	0.21898	0.46823	H134	0.63900	0.54142	0.01135
C63	0.61357	0.19307	0.49499	H135	0.61128	0.57882	-0.02757
C6	0.60194	0.12909	0.55404	H136	0.55057	0.55673	0.15590
C65	0.64290	0.15332	0.43372	H137	0.50886	0.55030	0.52633
C66	0.66286	0.19219	0.42051	H138	0.45998	0.39270	0.82087
C67	0.66332	0.38486	0.42295	H139	0.44120	0.45794	0.70598
C68	0.64441	0.40579	0.41802	H140	0.58522	0.05832	0.82651
C69	0.60645	0.39197	0.46416	H141	0.50980	0.04124	0.23749
C70	0.58979	0.35345	0.51391	H142	0.44937	0.01405	0.00010
C71	0.60889	0.33231	0.51530	H143	0.60672	0.02160	0.65143
N72	0.58894	0.41690	0.45041	H144	-3.43972	0.14858	1.54072

Table S3. Fractional atomic coordinates for the unit cell of TAPT-2,6-NA(OH)2 COF with A–A stacking.

Sample name: TAPT-2,6-NA(OH)2 COF							
Space group: P 1							
a = 42.3 Å, b = 42.1 Å, c = 3.5 Å, $\alpha = \beta = 90°$, $\gamma = 120°$							
R_{wp} = 6.84%, R_p = 3.66%							
**Atom**	*x*/*a*	*y*/*b*	*z*/*c*	**Atom**	*x*/*a*	*y*/*b*	*z*/*c*
C1	0.51283	1.05796	0.41180	N73	0.57952	0.52033	0.51742
C2	0.54531	1.05890	0.31681	C74	0.55051	0.51915	0.33988
C3	0.57823	1.09220	0.28517	C75	0.52573	0.53685	0.45991
C4	0.44732	1.02539	0.55203	C76	0.53081	0.57417	0.39829
C5	0.47796	1.02187	0.45825	C77	0.50080	0.58316	0.43884
C6	0.47203	0.98085	0.42146	C78	0.46672	0.55418	0.53871
O7	0.41336	0.99401	0.60989	C79	0.46178	0.51918	0.61103
N8	0.43964	0.94908	0.45601	C80	0.48999	0.51057	0.57306
C9	0.42433	0.90765	0.44241	C81	0.56498	0.60286	0.29795
C10	0.38592	0.88345	0.47343	C82	0.57102	0.63853	0.26387
C11	0.36833	0.84377	0.46916	C83	0.54361	0.64767	0.31521
C12	0.38751	0.82399	0.43601	C84	0.50635	0.62086	0.38073
C13	0.42642	0.84827	0.40562	C85	0.47898	0.63682	0.30746
C14	0.44419	0.88848	0.40777	O86	0.48239	0.47476	0.64526
C15	0.36668	0.77953	0.43490	O87	0.55518	0.68506	0.29485
N16	0.32858	0.75886	0.44049	N88	0.44978	0.63373	0.49677
C17	0.30907	0.72064	0.44114	N89	0.58825	0.20358	0.40543
N18	0.32965	0.70310	0.44033	C90	0.55627	0.17165	0.45533
C19	0.36773	0.72163	0.43517	C91	0.55064	0.13065	0.42278
N20	0.38539	0.75996	0.43044	C92	0.51556	0.09452	0.45992
C21	0.38981	0.69930	0.43845	C93	0.58155	0.12668	0.34868
C22	0.26484	0.69746	0.43992	C94	0.48289	0.09347	0.54808
C23	0.37317	0.66127	0.53734	C95	0.45016	0.06013	0.59287
C24	0.39255	0.64082	0.54995	O96	0.61708	0.15635	0.34545
C25	0.43015	0.65584	0.45232	H97	0.54582	1.03383	0.26864
C26	0.44709	0.69381	0.34947	H98	0.60292	1.09159	0.22006
C27	0.42784	0.71464	0.34595	H99	0.49587	0.97854	0.36464
C28	0.24108	0.71267	0.47956	H100	0.39398	0.99896	0.71536
C29	0.20104	0.69077	0.47726	H101	0.36899	0.89588	0.50264
C30	0.18154	0.65179	0.43154	H102	0.33890	0.82821	0.49494
C31	0.20522	0.63699	0.39074	H103	0.44351	0.83600	0.37871
C32	0.24476	0.65854	0.39644	H104	0.47357	0.90442	0.38351
N33	0.14019	0.62620	0.41884	H105	0.34460	0.64707	0.61590
C34	0.10880	0.62719	0.46676	H106	0.37775	0.61248	0.64005
C35	0.06766	0.59241	0.43540	H107	0.47580	0.70794	0.27785
C36	0.03214	0.59244	0.47058	H108	0.44305	0.74330	0.26640
C37	-0.00462	0.55909	0.42348	H109	0.25364	0.74205	0.51494
C38	-0.00448	0.52695	0.33130	H110	0.18547	0.70458	0.51017
C39	0.02825	0.52632	0.30164	H111	0.19257	0.60763	0.35294
C40	0.06290	0.55702	0.36445	H112	0.25994	0.64429	0.36274
C41	0.03189	0.62467	0.55586	H113	0.11152	0.65344	0.53453
O42	0.09190	0.55014	0.36390	H114	-0.02975	0.50164	0.28401
C43	0.99911	0.62507	0.59887	H115	0.02702	0.50057	0.23889
C44	0.96418	0.59362	0.55903	H116	0.05718	0.65015	0.59548

C45	0.95989	0.55894	0.46815	H117	0.10955	0.56318	0.58110
C46	0.91874	0.52447	0.43195	H118	1.00092	0.65094	0.66832
O47	0.93339	0.59719	0.61487	H119	0.91583	0.49793	0.37621
N48	0.88754	0.52603	0.46582	H120	0.93888	0.62196	0.71530
C49	0.84620	0.50057	0.45209	H121	0.83542	0.54501	0.51433
C50	0.82262	0.51556	0.48399	H122	0.76795	0.50847	0.50558
C51	0.78306	0.49408	0.47914	H123	0.77393	0.41023	0.38454
C52	0.76289	0.45507	0.44427	H124	0.84207	0.44750	0.39065
C53	0.78656	0.43969	0.41294	H125	0.58434	0.31653	0.50921
C54	0.82660	0.46148	0.41583	H126	0.55441	0.24809	0.50354
C55	0.71865	0.43195	0.44141	H127	0.65865	0.25702	0.33304
N56	0.69812	0.44954	0.44826	H128	0.68859	0.32459	0.34364
C57	0.66004	0.43107	0.44315	H129	0.68374	0.50560	0.62355
N58	0.64231	0.39274	0.43544	H130	0.65118	0.54068	0.65087
C59	0.66099	0.37312	0.43208	H131	0.55231	0.44583	0.29538
N60	0.69910	0.39376	0.43303	H132	0.58457	0.40986	0.27977
C61	0.64014	0.32867	0.42706	H133	0.53706	0.49773	0.12807
C62	0.63811	0.45356	0.44886	H134	0.44388	0.55885	0.57298
C63	0.60136	0.30433	0.47016	H135	0.43492	0.49778	0.69658
C64	0.58367	0.26411	0.46755	H136	0.58761	0.59795	0.25833
C65	0.60346	0.24503	0.41817	H137	0.59826	0.66063	0.20079
C66	0.64173	0.26930	0.37375	H138	0.48947	0.65791	0.09095
C67	0.65926	0.30897	0.37975	H139	0.45694	0.45795	0.74809
C68	0.65512	0.49161	0.54685	H140	0.54083	0.69266	0.46670
C69	0.63609	0.51235	0.56098	H141	0.53256	0.17378	0.52579
C70	0.59848	0.49769	0.46603	H142	0.48222	0.11856	0.58863
C71	0.58109	0.45969	0.36397	H143	0.42591	0.06132	0.66455
C72	0.60005	0.43853	0.35855	H144	0.62157	0.17387	0.56432

S8. FTIR Spectral Profiles of Monomers and COFs

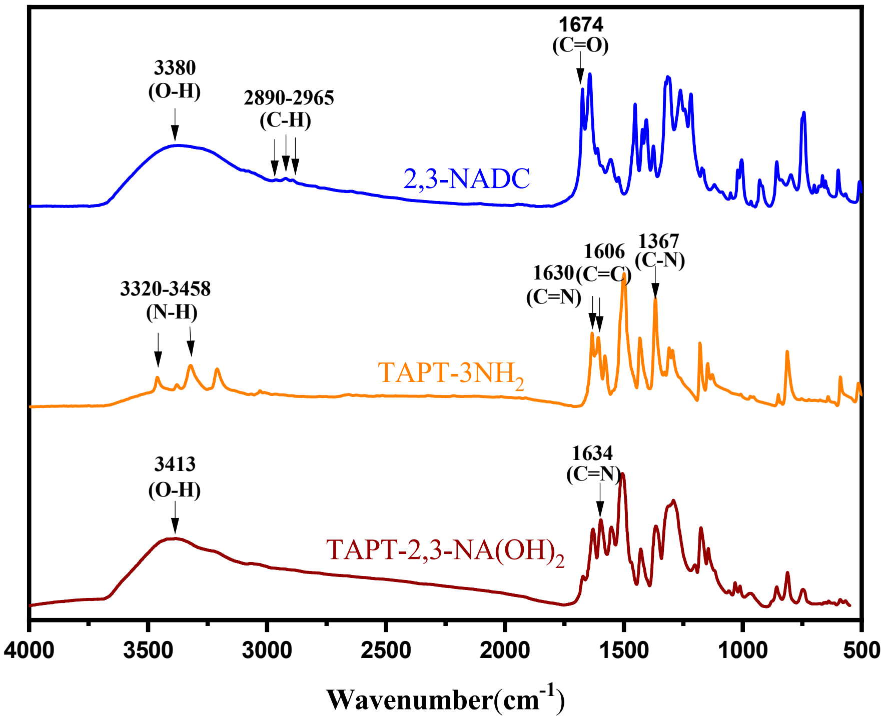

Figure S13. FTIR spectrum of 2,3-NADC, TAPT-3NH$_2$, and TAPT-2,3-NA(OH)$_2$ COFs.

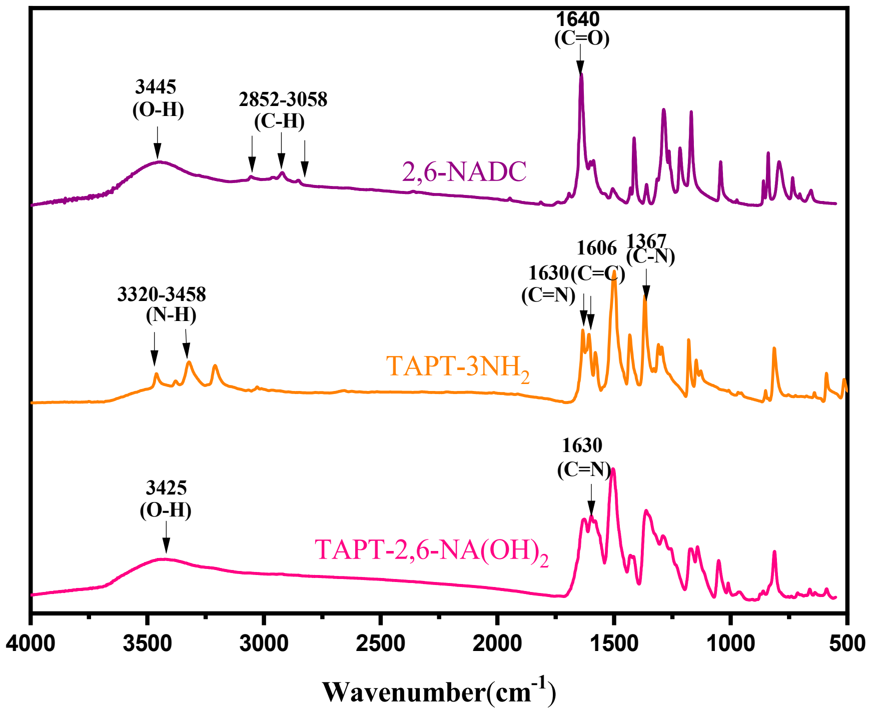

Figure S14. FTIR spectrum of 2,6-NADC, TAPT-3NH2, and TAPT-2,6-NA(OH)2 COFs.

S9. X-Ray Photoelectron Spectroscopy Analysis

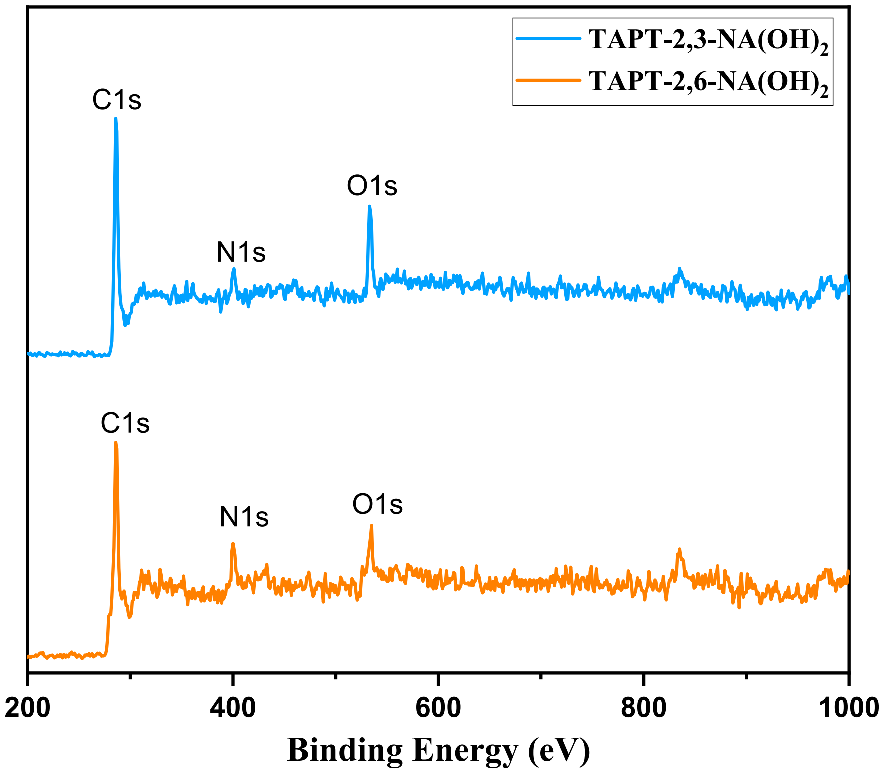

Figure S15. XPS spectra of TAPT-2,3-NA(OH) and TAPT-2,6-NA(OH)2 COFs.

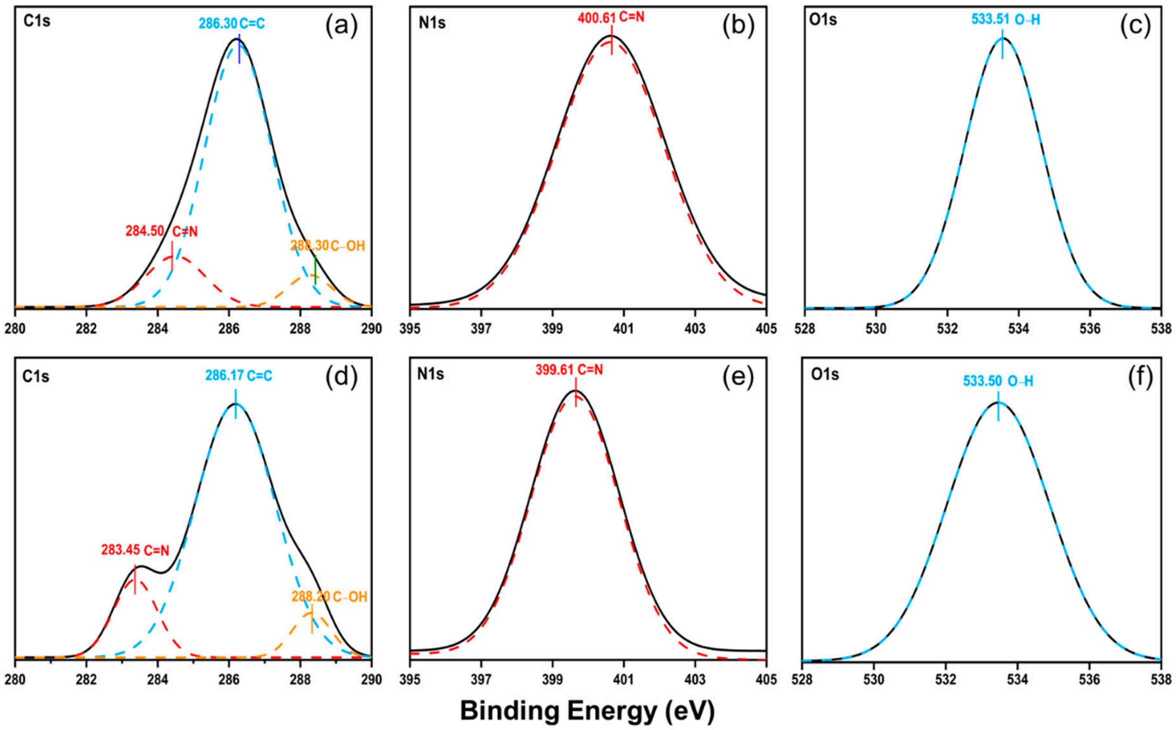

Figure S16. XPS spectra of the (a,d) C1s, (b,e) N1s and (c,f) O1s peaks for (a–c) TAPT-2,3-NA(OH) and (d–f) TAPT-2,6-NA(OH)$_2$ COFs.

Table S4. XPS fitting positions of TAPT-2,3-NA(OH)$_2$ and TAPT-2,6-NA(OH)$_2$ COF.

Sample		C species			N species	O species
		C=N	C=C	C–OH	=N–	–O–H
COFs	TAPT-2,3-NA(OH)$_2$	284.60	286.30	288.30	400.61	533.51
	TAPT-2,6-NA(OH)$_2$	283.45	286.17	288.20	399.61	533.50

Table S5. XPS fitting ratio of TAPT-2,3-NA(OH)$_2$ and TAPT-2,6-NA(OH)$_2$ COF.

Sample		C species			N species	O species
		C=N	C=C	C–OH	=N–	–O–H
COFs	TAPT-2,3-NA(OH)$_2$	13.78	79.82	6.40	100	100
	TAPT-2,6-NA(OH)$_2$	13.37	79.82	6.81	100	100

S10. Transmission Electron Microscopy (TEM)

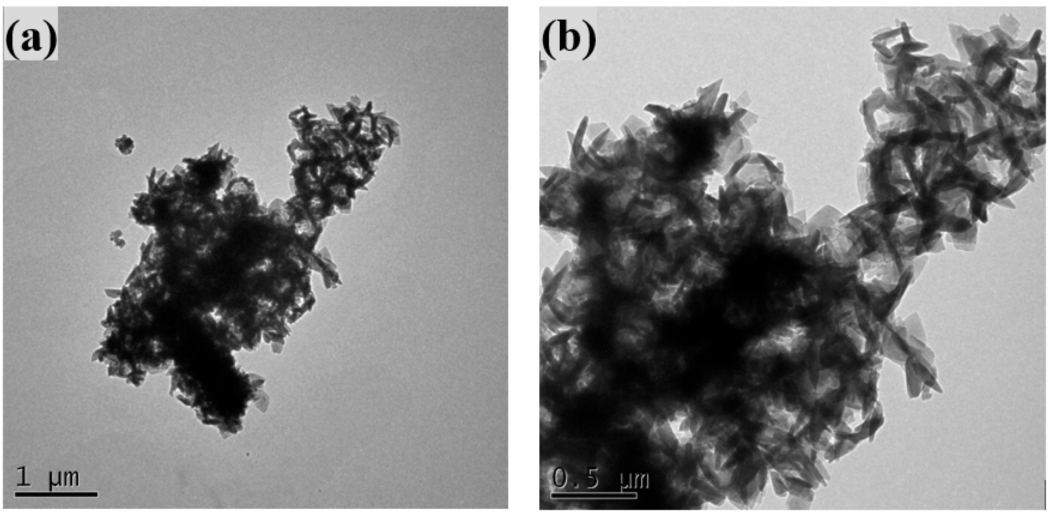

Figure S17. TEM images of the TAPT-2,3-NA$(OH)_2$ COF recorded at various magnifications: (a) 1 μm, (b) 0.5 μm.

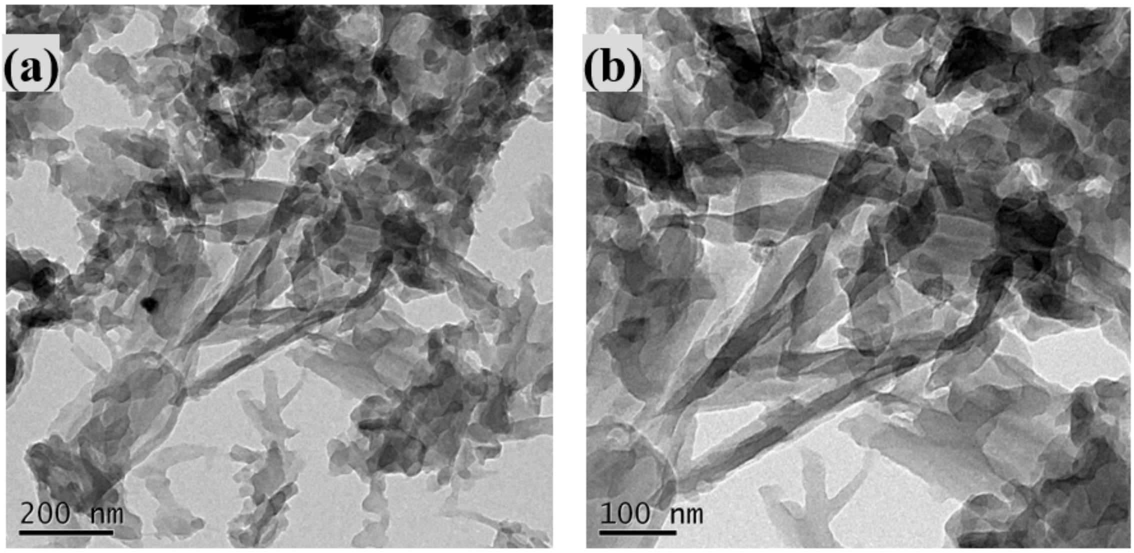

Figure S18. TEM images of the TAPT-2,6-NA$(OH)_2$ COF recorded at various magnifications: (a) 200 nm, (b) 100 nm.

S11. Field Emission Scanning Electron Microscopy (FE-SEM)

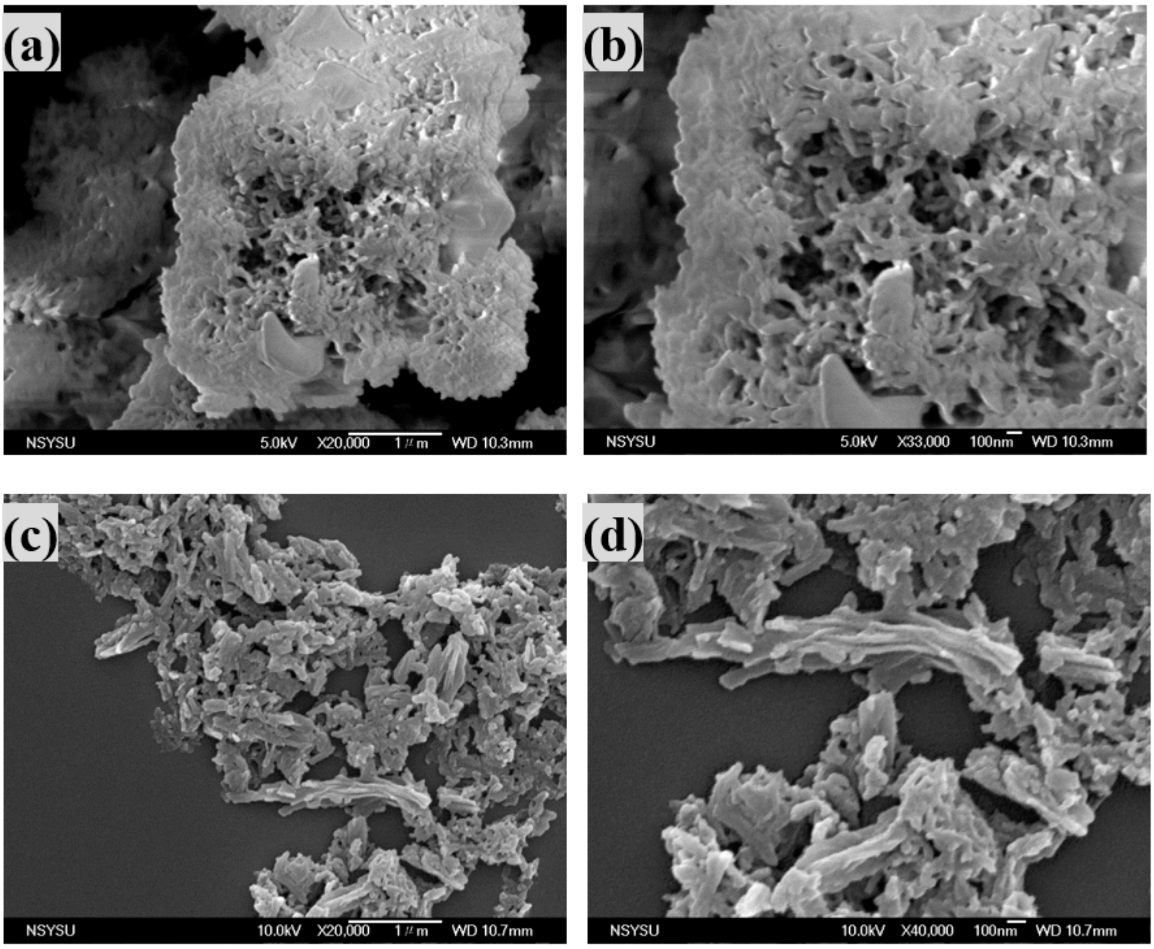

Figure S19. FE-SEM images of the (a, b) TAPT-2,3-NA$(OH)_2$ COF, (c, d) TAPT-2,6-NA$(OH)_2$ COF, recorded at various magnifications: (a, c) 1μm and (b, d) 100 nm.

S12. Thermal Gravimetric Analysis

Table S6. Values of $T_{d10\%}$, and Char yield of TAPT-2,3-NA$(OH)_2$ and TAPT-2,6-NA$(OH)_2$ COFs.

COF	$T_{d10\%}$ (°C)	Char yield (wt%)
TAPT-2,3-NA$(OH)_2$	435	60.44
TAPT-2,6-NA$(OH)_2$	460	60.07

S13. Electrochemical analysis

Working electrode cleaning: Prior to use, the glassy carbon electrode (GCE) was polished several times with 0.05-μm alumina powder, washed with EtOH after each polishing step, cleaned via sonication (5 min) in a water bath, washed with EtOH, and then dried in air.

Electrochemical Characterization: The electrochemical experiments were measured in a three-electrode cell using an Autolab potentiostat (PGSTAT204) in aqueous electrolyte 1 M KOH. The GCE was used as the working electrode (diameter: 5.61 mm; 0.2475 cm^2). A platinum wire was used as the counter electrode; Hg/HgO (RE-61AP, BAS) was used as the reference electrode. All reported potentials refer to the Hg/HgO potential. The GCE was modified with COF slurries as described elsewhere with some modifications.[S1-S3] The slurries were prepared by dispersing COF (45 wt. %), carbon black (45 wt. %), and Nafion (10 wt. %) in 2 mL of ethanol and sonicated for one hour. A portion of this slurry (10 μL) was pipetted onto the tip of the electrode which was then dried in air for 30 minutes before use. The electrochemical performance was studied using cyclic voltammetry (CV) at different sweep rates from 5 to 200 mV s^{-1}, galvanostatic charge–discharge (GCD) in the potential range from 0.18 to –0.92 V vs. Hg/HgO at different current densities from 2 to 20 A g^{-1} in a 1 M KOH aqueous electrolyte solution.

The specific capacitance was calculated from galvanostatic charge–discharge experiments using the following equation:[S3,S4]

$$Cs = (I\Delta t)/(m\Delta V) \quad \text{(S1)}$$

Where Cs (F/g) is specific capacitance of the supercapacitor, I (A) is the discharge current, ΔV (V) is the potential window, Δt (s) is the discharge time, and m (g) is the mass of COF on the electrode.

The energy density (E, Wh/kg), and the power density (P, W/kg) were calculated using
the following equations:

$$E = 1000C(\Delta V)^2/(2*3600) \quad \text{(S2)}$$

$$P = E/(t/3600) \quad \text{(S3)}$$

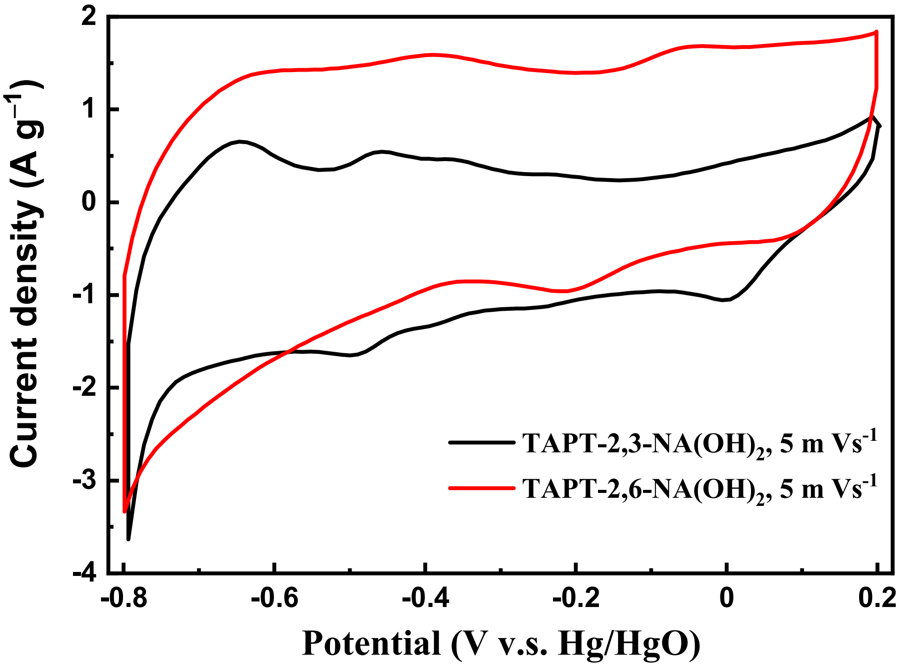

Figure S20. Comparison CV curves of the TAPT-2,3-NA$(OH)_2$ and TAPT-2,6-NA$(OH)_2$ COFs, measured at a scan rate (5 mV s^{-1}).

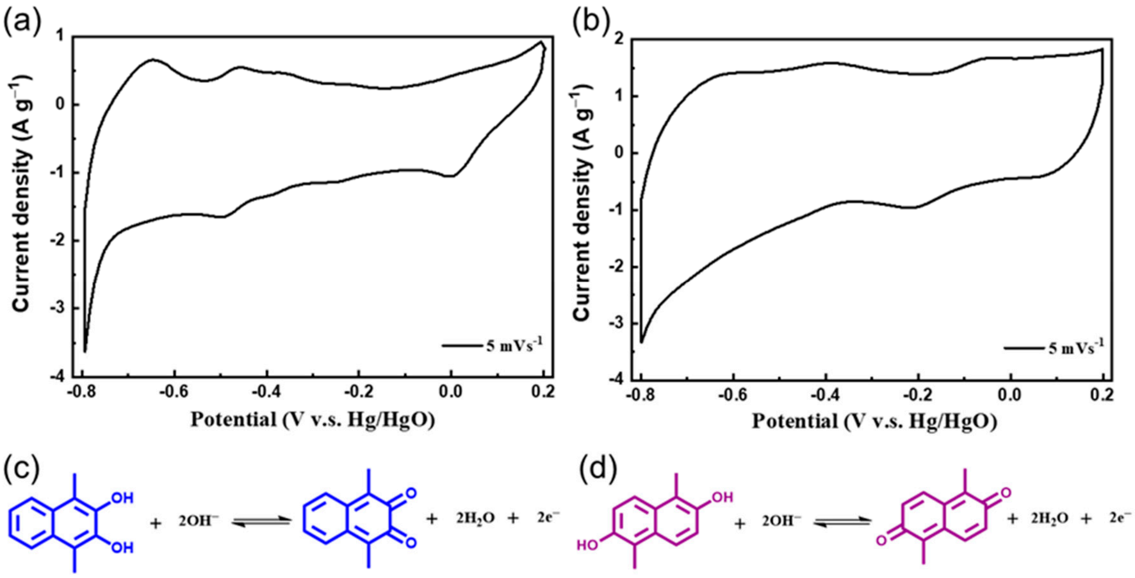

Figure S21. CV curves of the (a) TAPT-2,3-NA(OH)2 and (b) TAPT-2,6-NA(OH)2 COFs, measured at a scan rate (5 mV s^{-1}). Redox transformation of the (c) TAPT-2,3-NA(OH)2 COF, (d) TAPT-2,6-NA(OH)2 COF.

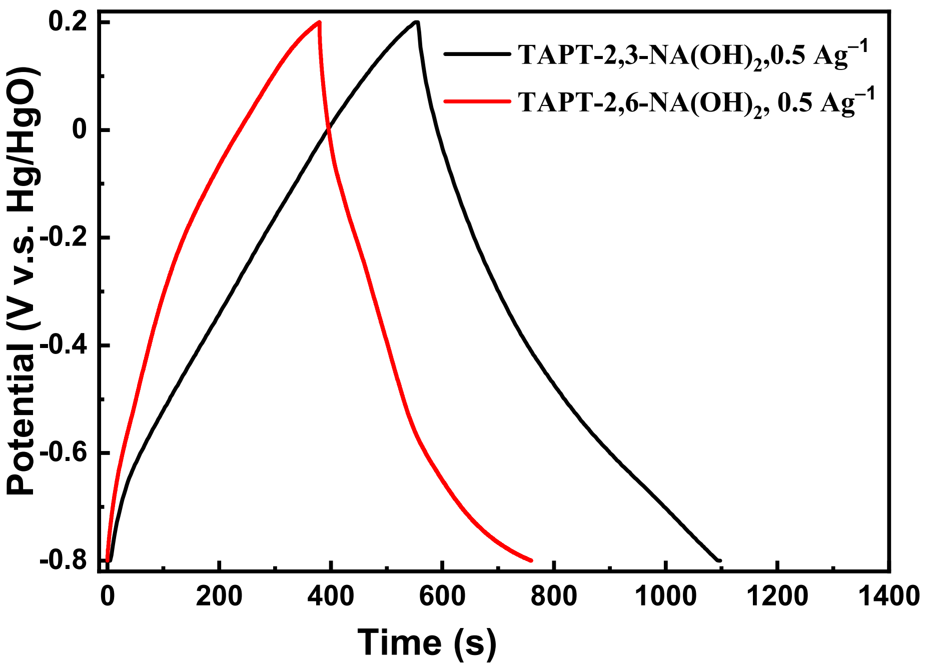

Figure S22. GCD curve of TAPT-2,3-NA(OH)2 and TAPT-2,6-NA(OH)2 COFs, measured at a current density (0.5 A g^{-1}).

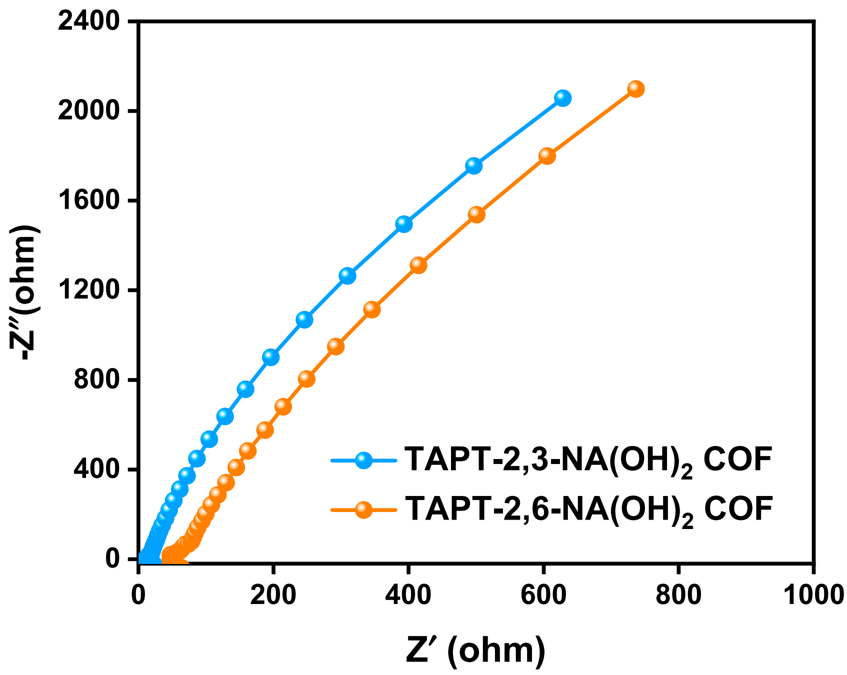

Figure S23. Electrochemical impedance spectroscopy (Nyquist plots) of TAPT-2,3-NA(OH)$_2$ and TAPT-2,6-NA(OH)$_2$ COFs.

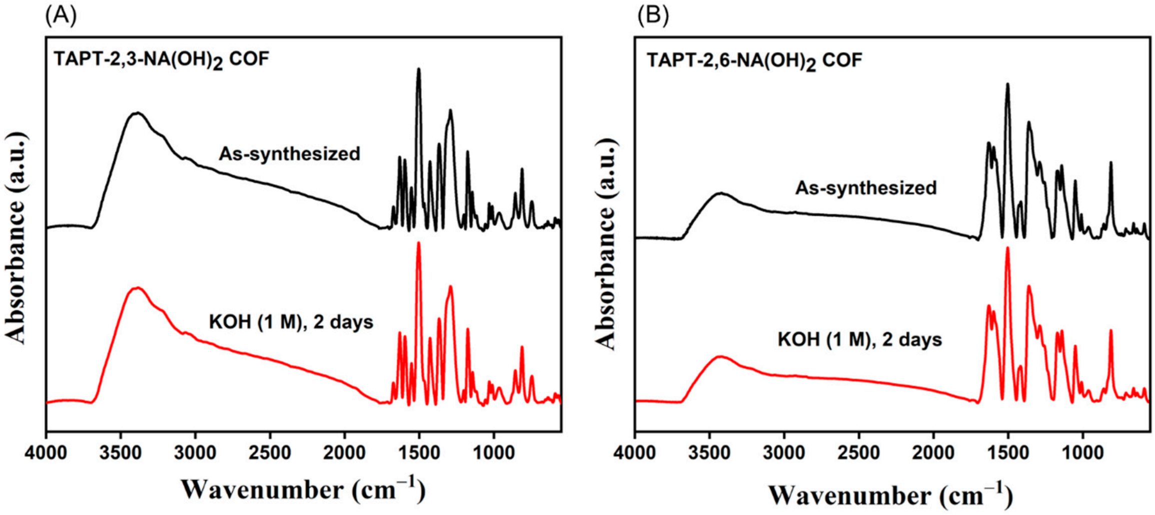

Figure S24. FTIR spectra of TAPT-2,3-NA(OH)$_2$ and TAPT-2,6-NA(OH)$_2$ COFs as-synthesized and after soaking 2 days in KOH (1 M).

S14. Comparison Study

Table S7. Comparison between the specific surface area/specific capacitance of TAPT-2,3-NA(OH)$_2$ and (b) TAPT-2,3-NA(OH)$_2$ COFs with those of previously reported COFs for supercapacitor application.

COFs	S_{BET} (m^2 g^{-1})	Capacitance	Ref.
TPT-DAHQ	1855	256 F g^{-1} at 0.5 A g^{-1}	S1
Car-TPA COF	1334	13.6 F g^{-1} at 0.2 A g^{-1}	S2
Car-TPP COF	743	14.5 F g^{-1} at 0.2 A g^{-1}	S2
Car-TPT COF	721	17.4 F g^{-1} at 0.2 A g^{-1}	S2
DAAQ-TFP COF	1280	48 ± 10 F g^{-1} at 0.1 A g^{-1}	S3
TPA–COF-1	714	51.3 F g^{-1} at 0.2 A g^{-1}	S5

TPA–COF-2	478	14.4 F g^{-1} at 0.2 A g^{-1}	S5
TPA–COF-3	557	5.1 F g^{-1} at 0.2 A g^{-1}	S5
TPT–COF-4	1132	2.4 F g^{-1} at 0.2 A g^{-1}	S5
TPT–COF-5	1747	0.34 F g^{-1} at 0.2 A g^{-1}	S5
TPT–COF-6	1535	0.24 F g^{-1} at 0.2 A g^{-1}	S5
TaPay-Py COF	687	209 F g^{-1} at 0.5 A g^{-1}	S6
DAB-TFP COF	385	98 F g^{-1} at 0.5 A g^{-1}	S6
TFP-TPA COF	457	291.1 F g^{-1} at 2 A g^{-1}	S7
TFP-TPP COF	686	185.5 F g^{-1} at 2 A g^{-1}	S7
TFP-Car COF	362	149.3 F g^{-1} at 2 A g^{-1}	S7
TAPT-2,3-NA$(OH)_2$	429	271 F g^{-1} at 0.5 A g^{-1}	This work
TAPT-2,6-NA$(OH)_2$	1089	190 F g^{-1} at 0.5 A g^{-1}	This work

S15. References

S1. El-Mahdy, A. F. M.; Hung, Y. H.; Mansoure, T. H.; Yu, H. H.; Chen, T.; Kuo, S. W. A hollow microtubular triazine- and benzobisoxazole-based covalent organic framework presenting sponge-like shells that functions as a high-performance supercapacitor. *Chem. Asian J.* **2019**, *14*, 1429–1435.

S2. El-Mahdy, A. F. M.; Young, C.; Kim, J.; You, J.; Yamauchi, Y.; Kuo, S. W. Hollow Microspherical and Microtubular [3 + 3] Carbazole-Based Covalent Organic Frameworks and Their Gas and Energy Storage Applications. *ACS Appl. Mater. Interfaces* **2019**, *11*, 9343–9354.

S3. DeBlase, C. R.; Silberstein, K. E.; Truong, T.–T.; Abruña, H. D.; Dichtel, W. R.; β-Ketoenamine-Linked Covalent Organic Frameworks Capable of Pseudocapacitive Energy Storage. *J. Am. Chem. Soc.*, **2013**, *135*, 16821–16824.

S4. Alabadi, A.; Yang, X.; Dong, Z.; Li, Z.; Tan, B.; Nitrogen-doped activated carbons derived from a co-polymer for high supercapacitor performance. *J. Mater. Chem. A*, **2014**, *2*, 11697–11705.

S5. EL–Mahdy, A. F. M.; Kuo, C.–H.; Alshehri, A. Young, C.; Yamauchi, Y.; Kim, J.; Kuo, S.–W.; Strategic design of triphenylamine- and triphenyltriazine-based two-dimensional covalent organic frameworks for CO2 uptake and energy storage. *J. Mater. Chem. A*, ***2018***, *6*, 19532–19541.

S6. Khattak, A. M.; Ghazi, Z. A.; Liang,B.; Khan, N. A.; Iqbal, A.; Li, L.; Tang, Z. A redox-active 2D covalent organic framework with pyridine moieties capable of faradaic energy storage. *J. Mater. Chem. A*, ***2016***, *4*, 16312-16317.

S7. EL-Mahdy, A. F. M.; Hung, Y. -H.; Mansoure, T. H.; Yu, H. -H.; Hsu, Y. -S.; Wu, K. C. - W.; Kuo, S. -W. Synthesis of [3 + 3] β-ketoenamine-tethered covalent organic frameworks (COFs) for high-performance supercapacitance and CO_2 storage. *J. Taiwan Inst. Chem. Eng.* **2019**, *103*, 199–208.
